# Diachronic shifts in lithic technological transmission between the eastern Eurasian Steppe and northern China in the Late Pleistocene

**DOI:** 10.1371/journal.pone.0275162

**Published:** 2022-11-03

**Authors:** Chao Zhao, Youping Wang, John P. Walden

**Affiliations:** 1 School of History and Civilization, Shaanxi Normal University, Xi’an, China; 2 School of Archaeology and Museology, Peking University, Beijing, China; 3 Department of Anthropology, Harvard University, Boston, Massachusetts, United States of America; Griffith University, AUSTRALIA

## Abstract

The successful occupation of the eastern Eurasian Steppe in the Late Pleistocene improved cultural connections between western Eurasia and East Asia. We document multiple waves of lithic technological transmission between the eastern Eurasian Steppe and northern China during 50–11 cal. ka BP. These waves are apparent in the sequential appearance of three techno-complexes in northern China: (1) the Mousterian techno-complex, (2) the blade techno-complex mixed with Mousterian elements, (3) and the microlithized blade techno-complex. These lithic techno-complexes were transmitted under different paleoenvironmental conditions along different pathways through the eastern Eurasian Steppe. The Mousterian techno-complex and the blade techno-complex mixed with Mousterian elements were only dispersed in the north and west peripheries of northern China (50–33 cal. ka BP). We argue that these techno-complexes failed to penetrate into the hinterland of northern China because they were not well suited to local geographical conditions. In contrast, the microlithized blade technology which diffused from the eastern Eurasian Steppe was locally modified into a Microblade techno-complex which was highly suited to local environmental conditions, and proliferated across the hinterland of northern China (28/27-11 cal. ka BP). The subsequent spread of microblade technology over vast regions of Mongolia and Siberia indicates that the Pleistocene inhabitants of northern China not only adopted and modified technologies from their neighbors in the Eurasian Steppe, but these modified variants were subsequently transmitted back into the Eurasian Steppe. These episodes of technological transmission indicate complicated patterns of population dispersal and technological interaction across northern China and the eastern Eurasian Steppe.

## Introduction

Geographical obstacles and great distances impacted the cultural transmission of lithic technologies between western Eurasia and East Asia [1]. In contrast to the successive development of the core-flake, Acheulean, Mousterian, and blade complexes which are widely apparent in many parts of western Eurasia in the Paleolithic, the lithic development trajectory in East Asia reveals the persistence of the core-flake and cobble-chopper techno-complexes until the later phase of the Upper Paleolithic [2]. Even though the Acheulean lithic techno-complex can be identified in several regions of East Asia, potentially indicating convergent lithic evolution or punctuated lithic technological transmission with western Eurasia, the sites associated with the Acheulean techno-complex are far fewer and not as widely spread as those of the core-flake techno-complexes [3]. However, recent findings indicate that from around 50 ka BP, lithic assemblages from several sites in northern China (part of East Asia) show typical characteristics of western Eurasian lithic techno-complexes [4]. These include the Terminal Middle Paleolithic (TMP ~50–38 ka BP) Mousterian techno-complex, the Initial Upper Paleolithic (IUP ~43-32ka BP) blade techno-complex (mixed with the Mousterian technological elements), and the Middle Upper Paleolithic (MUP ~32–23 ka BP) blade techno-complex (characterized by microlithization). The latest research also indicates that the microblade techno-complex which occurred in the east part of northern China and its eastern surroudings from ~28 cal. ka BP and proliferated widely across northern China and eastern Eurasian Steppe during the Late Upper Paleotlihic (~23–11 ka BP) likely developed from modified microlithized blade technology introduced from the eastern Eurasian Steppe, moreso than the local core-flake tradition [5]. Such patterns suggest enhanced technological connections between western Eurasia and East Asia from 50 cal. ka BP.

Comparing the lithic developmental patterns between the eastern Eurasia Steppe and northern China is crucial for understanding the increased level of lithic technological connection between western Eurasia and East Asia at the end of the Paleolithic. The Eastern Eurasian Steppe is mainly composed of the Mongolia Plateau and the eastern part of South Siberia. It is located to the northeast of West and Central Asia, and borders west, north and east margins of northern China. Unlike the formidable desert and mountainous Inner Asian regions situated directly between western Eurasia and East Asia, the eastern Eurasian Steppe poses less geographical obstacles and could serve as a relatively convenient corridor of technological transmission between western Eurasia and East Asia in the Late Pleistocene [6]. The lithic techno-complexes here show strong similarities with the broad regions situated directly to the west, like Central Asia, West Asia, and East-Central Europe from the TMP to the MUP period. Hence the eastern Eurasian Steppe was likely the source area from which the lithic techno-complexes with western characteristics were transmitted to the east [7–9].

Based on the cross-regional comparison of lithic techno-complexes over time, we have identified multiple episodes of technological transmission between the eastern Eurasian Steppe and northern China at the end of the Late Pleistocene (approx. 50–11 cal. ka BP). Through synthetic and comparative studies of the characteristics and distributions of lithic remains associated with different waves of technological transmission under different paleoclimatic circumstances, we further examine the nature, spread and possible routes of these transmissions and evaluate their impacts on local cultural adaptation.

## Methods

Various methods were employed to reconstruct the transmission of lithic techno-complexes and contextualize these within broader climatic and environmental trends. Firstly, we temporally contextualize archaeological sites in the eastern Eurasian Steppe and northern China based on chronometric data and temporally diagnostic lithic features (S1 Table, S1 File). Secondly, a comprehensive lithic analysis is conducted to investigate the specific characteristics of lithic remains from each site and associate them with different prevailing techno-complexes found in the eastern Eurasian Steppe and northern China during 50–11 cal. ka BP, including the indigenous core-flake techno-complex of northern China, the TMP Mousterian techno-complex, IUP blade techno-complex mixed with Mousterian traces, and the MUP microlithized blade techno-complex, blade and initial microblade coexisted techno-complex, and the LUP (Late Upper Paleolithic) Microblade techno-complex (S1 Table, S1 File). These techno-complexes and the spatial distribution of sites associated with them is discussed in depth below. By examining the spread of different lithic techno-complexes over time we evaluate how different regions were impacted by each episode of cultural transmission and identify the direction and route of these transmissions.

To understand the mechanisms underlying different waves of lithic technological transmission and identify why each episode of transmission differed, we reconstruct the respective paleoenvironmental context associated with each major transmission. We then examine technological organization to identify the conditions and human responses which prompted changes in lithic technological transmission. Paleoenvironmental reconstruction is based on climate proxies derived from loess, stalagmites, and pollen. Investigation of technological organization is based upon the selection and integration of strategies for making, using, transporting, and discarding lithic tools and materials [10]. Our examination of technological organization takes into account economic concerns relative to environmental conditions and how these manifested in design and distribution of the specific lithic techno-complex.

## Spatial-temporal and paleoenvironmental context

**Fig 1:**
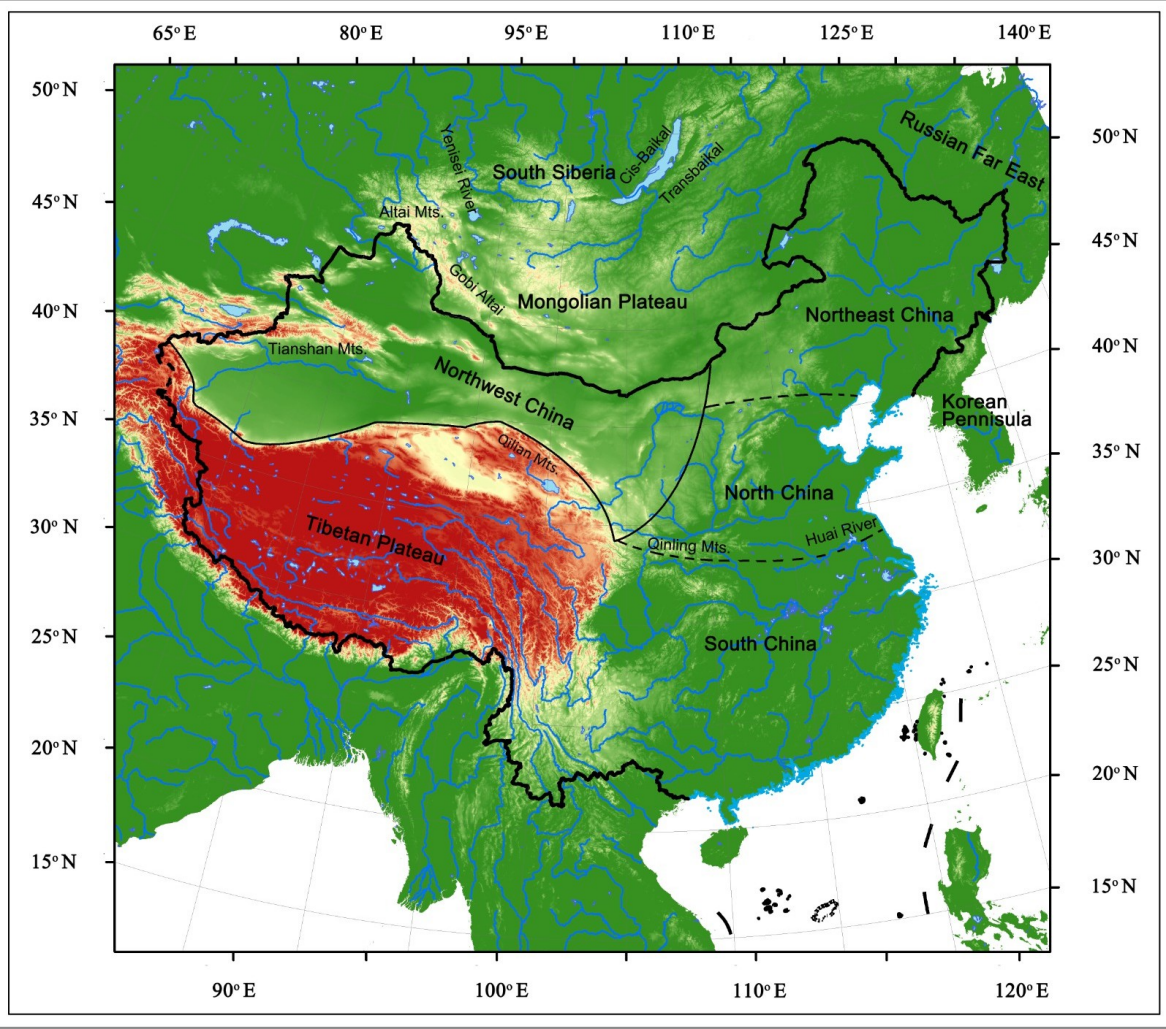
Map showing the location and topographic features of the subdivisions of northern China (Northeast China, Northwest China and North China) and its surroundings. The base map raster is made from the USGS digital elevation map data from http://earthexplorer.usgs.gov/.

For the purposes of this article, the eastern Eurasian Steppe refers specifically to the areas east of the Altai Mountains, including the Mongolian Plateau and regions across South Siberia, like the Altai Mountainous region, the Yenisei River valley, Cis-Baikal, and the Transbaikal regions. We define Northern China as the area encompassing the modern territory of China, the eastern part being north of the Qinling Mountain- Huai River line and the western part being north of the Tibetan Plateau (Fig 1). To facilitate analysis and discussion, northern China is further sub-divided into three regions in this paper, namely Northwest China, North China, and Northeast China (Fig 1). As for the intra-environmental variation across different parts of northern China, the weather becomes drier moving from the southeast to the northwest, with the decline in strength of the summer monsoon. In general, the climate becomes colder moving from south to north. Although this is somewhat complicated at the local level by other factors such as elevation and distance from the coast [11].

Of these regions, Northwest and Northeast China are closer to the eastern Eurasian Steppe than North China. The climate in Northwest China is dry because it is situated largely outside of the monsoon climate zone [11]. The climate in Northeast China is relatively colder due to its higher latitude and proximity to the Mongol-Siberia high pressure system. Humidity increases when moving west to east across Northeast China alongside the severity of the summer monsoon [11]. North China generally has a milder climate than Northwest and Northeast China. While the Tibetan Plateau is not included in the spatial scope of northern China in this paper, the discoveries there will also be briefly mentioned as they convey important information about cross-regional lithic technological transmission relevant with northern China.

Our main temporal focus is the end of the Late Pleistocene, ranging from about 50 to 11 cal. ka BP. This period is temporally divided into several periods and there is no unified scheme applied both in the eastern Eurasia Steppe and northern China. In northern China, the Upper Paleolithic is often divided into two phases by a time line between 30 and 20 cal. ka BP [12, 13], but the distinction between the Middle and Upper Paleolithic is more ambiguous and disputed [14]. In the eastern Eurasian Steppe, this period is often divided into the TMP and three successive periods of Upper Paleolithic from early to late, namely IUP, MUP, and LUP [15, 16]. Each of the periods corresponds with a diagnostic and different lithic techno-complex [17]. We analytically apply the “TMP-IUP-MUP-LUP” temporal scheme in this paper because it is less disputed and is heuristically useful for tracing diachronic shifts in lithic transmission between the eastern Eurasian Steppe and northern China.

The waves of technological transmission between the eastern Eurasian Steppe and northern China which are our focus occurred during two different paleoclimatic stages: MIS 3 (marine oxygen-isotope stage 3, ~57–29 cal ka BP) and MIS 2 (marine oxygen-isotope stage 2, ~29–15 cal ka BP). MIS 3 was an interglacial period in which conditions in the northern hemisphere were warmer and wetter than those during the preceding and following glacial maxima periods. The eastern Eurasian Steppe is part of the Mammoth Steppe biome, which is composed of steppe and patchily distributed woodlands [18]. The climatic conditions in northern China at this time varied regionally. This variation differs from the trans-regional climatic patterns today [19]. Generally, the climate in the eastern part of northern China (including North and Northeast China) was drier than today whereas the western part (including Northwest China) was more humid than today [20–22]. MIS 3 differences in humidity between the western and eastern parts of northern China were smaller than present-day conditions [23, 24]. The monsoon-effected region of northern China had a humid steppe biome with patchily distributed woodlands [21, 25]. The vegetation coverage was relatively lusher and more diverse than the proceeding LGM (Last Glacial Maximum) period [24, 26]. In the western part of northern China where the effects of the summer monsoon were limited, desert and steppe-desert proliferated in low lying areas. These regions contained chains of oases, and generally lake levels were higher than today meaning these oases could provide substantial amounts of water [20, 23, 27]. The western part of northern China also had more expansive mountainous forests than today, which extended below the elevation of the modern tree-line [28].

In contrast, MIS 2 comprises the Last Glacial Maximum and the beginning of the Post-Glacial period. The climate of the northern Hemisphere in this period was drier and colder than today. The tundra area in the eastern Eurasian Steppe was larger. The Mammoth Steppe biozone was more open and homogeneous with less patches of temperate forest and therefore less biodiversity [18]. In northern China, pollen analysis revealed a decrease of xylophyta species, suggesting earlier forests receded in this region in a similar manner as the Mammoth Steppe biozone [25, 29]. Moreover, a decrease in aquatic species and an increase in arid-tolerant species were identified in pollen profiles sampled in inner parts of northern China (including Northwest China and western Northeast China) [22, 30]. North and Northeast China became dominated by meadow-steppe, steppe, and desert-steppe during this time and the northern part of Northeast China became tundra around this time [31, 32]; MIS 2 vegetation coverage was less abundant and diverse than the MIS 3 period [26, 33–35]. Generally, the extent of humidity and temperature decrease varied between the different sub-regions. The higher-latitude regions experienced more pronounced temperature decreases than lower-latitude areas [36]. North and Northeast China exhibited a more pronounced decrease in humidity than Northwest China [37].

## Results

### Human occupation of the eastern Eurasian Steppe and its impacts on cultural interaction between western Eurasia and northern China

The eastern Eurasian Steppe was likely occupied comparatively later than neighboring parts of western Eurasia and northern China as it was a challenging adaptive environment. Situated in the mid-to-high latitudes of the continental northern hemisphere, the eastern Eurasian Steppe has a fluctuating climate with extreme seasonality. The short growing seasons, long and cold winter, and fewer edible plant resources poses serious challenges to survival. People could not permanently occupy this area until they had developed efficient means to cope with these challenges, these means took time to develop [38].

The oldest known site in the eastern Eurasian Steppe is the Karama site located in the Altai Mountains, which dates to 800 kya [39]. However, Lower Paleolithic (prior to 200 kya) sites are rare in the eastern Eurasian Steppe. Some of these Lower Paleolithic sites have questionable artifacts or lack clear stratigraphy [40]. Therefore, while early humans spread into this region in the Middle Pleistocene, evidence does not yet suggest consistent occupations at this time.

Archaeological evidence of human occupations is more abundant in the eastern Eurasian Steppe from the Middle Paleolithic period. The Middle Paleolithic sites in this area are mainly distributed in the Altai Mountains of South Siberia and the western and central parts of the Mongolian Plateau. In the subsequent Upper Paleolithic period, the number of sites increased and these became more widely distributed across the landscape. Regions which were devoid of any Middle Paleolithic occupation became infilled with Upper Paleolithic occupations, indicating population increase and further eastward expansion into the Eurasian Steppe [41, 42]. Middle Paleolithic lithic remains in the eastern Eurasian Steppe are associated with the Mousterian techno-complex, which is followed by the spread of blade technology in the IUP and MUP periods [43].

The increased intensity of occupation in the eastern Eurasian Steppe by the Upper Paleolithic is closely associated with technologies which people developed to adapt to the high-latitude environment. Tools associated with the Mousterian and Blade techno-complexes increased hunting efficiency by providing regularized formal end products [44]. Moreover, the discoveries of bone needles and awls at the beginning of the Upper Paleolithic [45] and the semi-subterranean dwelling structures at Upper Paleolithic sites in South Siberia [46, 47] suggested that people had developed efficient strategies (like clothes, cold-proof dwellings) to cope with the challenges associated with cold environments. Human adaptation to, and successful occupation of the eastern Eurasian Steppe profoundly changed patterns of cultural interaction between western Eurasia and northern China. Previously unoccupied, or sporadically occupied areas to the north of northern China were now occupied, which facilitated communication and the flow of people, ideas and material culture.

### Technological transmission between northern China and the eastern Eurasian Steppe during the TMP and IUP Periods

#### The TMP and IUP techno-complex in the eastern Eurasian Steppe

The Middle Paleolithic remains in the eastern Eurasian Steppe are characteristic of the Mousterian techno-complex found in Central and West Asia [48, 49]. Only a limited number of Middle Paleolithic remains have been chronometrically dated. The lowest cultural layer at Denisova Cave in the Altai Mountains is estimated to be occupied during the Kazantsev interglacial or early Zyrain glacial (130–80 ka BP) periods, based on stratigraphic position and correlation with the pollen spectrum [40]. Most other Middle Paleolithic occupations in the Altai Mountains were dated to ~70–40 ka BP [50, 51]. The only chronometric data from specific stratigraphic contexts in Mongolia comes from Layer 6 of Kharganyn Gol 5 (located in north-central Mongolia) which was dated to 50–45 cal. ka BP [52].

IUP remains were stratigraphically situated above the TMP techno-complex at multi-component sites in Siberia and Mongolia. The earliest layer of IUP techno-complex materials from the Kara-Bom site in the Altai Mountains dates to ~43 ka BP [53]. Nonetheless, strata at some sites in Siberia with classic Middle Paleolithic Mousterian assemblages date to as late as 40–30 cal. ka BP [54]. This longevity shows that the typical Mousterian techno-complex persisted alongside the IUP lithic assemblages for a long time in this region. The striking difference between the TMP and the IUP was the prevalence of blades [16]. IUP blades were slender, laminar pieces which were systematically flaked from pre-formed cores. Blades were sometimes used as blanks and retouched along the edges to be processed into various tools, such as scrapers, end scrapers, burins, and points [55].

The appearance of blade technology was once considered to be a cultural marker of modern human behavior [56]. Although recent discoveries have challenged the view that blade technology was unique to modern humans alone, the consistent widespread prevalence of the blade techno-complex is still regarded as a defining characteristic of the Upper Paleolithic in western Eurasia, which is often but not exclusively associated with modern humans [55]. The IUP techno-complex represented an admixture of emergent blade technologies and elements inherited from the Mousterian tradition. Blades were not only produced from prismatic or sub-prismatic cores (which are considered the typical blade production method during the Upper Paleolithic), but were also manufactured from Levallois flat-faced cores. Other Levallois reduction methods used for producing elongated flakes or regular points, typical in the TMP Mousterian techno-complex, also persisted in the IUP techno-complex. In terms of lithic toolkit composition, emergent tool types like end scrapers and burins are often found alongside traditional Mousterian tools like side scrapers and denticulates [57].

The IUP techno-complex was a widespread cultural phenomenon distributed across several regions of East-Central Europe, West Asia, Central Asia, and the eastern Eurasian Steppe. Despite beginning later in the eastern Eurasian Steppe than Central and West Asia, the IUP techno-complex lasted longer in this region, with IUP remains dating to as late as ~30 cal. ka BP [57]. The IUP lithic remains in the Russian Altai and the Gobi-Altai in Mongolia show stronger influences of the TMP Mousterian traditions. This TMP Mousterian influence is apparent in high proportions of traditional Mousterian tools and the widespread application of the Levallois technique [16, 54]. In comparison, sites located in North Mongolia and the Transbaikal region show limited evidence of Mousterian assemblages [16]. Blades were primarily produced using prismatic cores not Levallois cores. Some of the sites earlier than 30 cal. ka BP in North Mongolia and the Transbaikal region were devoid of any artifacts associated with the Mousterian tradition [58].

#### The TMP techno-complex in northern China

**Fig 2:**
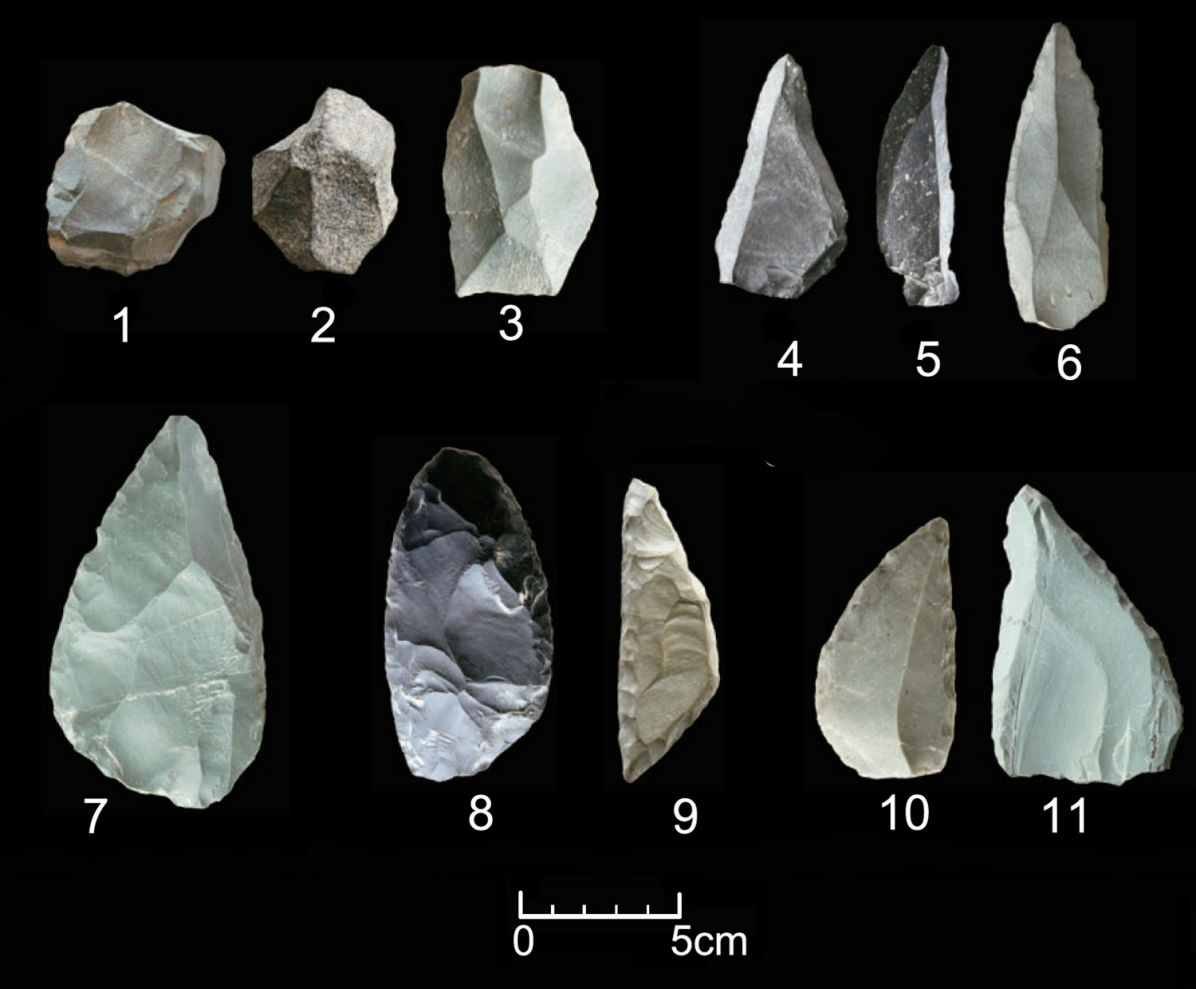
Typical artifacts of the TMP techno-complex found in the Tongtiandong Cave site of Northwest China. (1: Levallois core; 2: Discoid core; 3: Levallois flake; 4,5,6: Levallois points; 7,10,11: Mousterian point; 8,9: Scraper; 1–11: after [60]).

The typical Mousterian techno-complex was long considered absent from China due to a lack of convincing evidence [59]. However, recent discoveries reveal unequivocal evidence of this techno-complex distributed sporadically across peripheral areas of Northwest and Northeast China. At Tongtiandong Cave in Northwest China, the whole reduction sequence of the Mousterian techno-complex has been identified. This sequence comprised Levallois flake cores, Levallois flakes, points, and denticulates (Fig 2) [60]. The Tongtiandong Cave site is located in the Xinjiang Autonomous Region immediately south of the Altai Mountains. The Mousterian cultural layer was radiocarbon dated to approximately 46–44 cal. ka BP [60].

In Northeast China, the Mousterian techno-complex was also identified at the Jinsitai and Sanlongdong caves. A typical Levallois core, a few Levallois flakes and points, and a considerable amount of the debitage associated with Levallois reduction sequences were recently found at the Jinsitai cave site [61]. In association with these diagnostic Mousterian remains was an abundance of cores, flakes, and tools showing expedient core-flake production of the type widely distributed across the hinterland of northern China at this time [62]. Cultural Layers 7 and 8 at Jinsitai cave show a combination of simple flake-core technology and Mousterian technology. While radiocarbon dates reveal a long temporal occupational sequence (seen Table 1), two charcoal samples are likely intrusive from an upper cultural layer. Removal of these outliers due to contamination concerns, suggests that Layers 7 and 8 mainly span from 47–38 cal. ka BP [61].

**Table 1:** Chronological information about the MP and IUP techno-complex sites in northern China. The radiocarbon data was calibrated by using OxCal 4.4 and IntCal20 calibration curve.

Site	Location	Layer/	Dated	Dating	Lab	Date		Reference
		Context	Material	Method	No.	Uncal. BP	Cal. BP	
Tongtiandong Cave (TMP)	47°10’45.9"N, 86°8’11.3"E	6A-9	Bone	AMS ^14^C	BA160938	40950±260	44469–43286	[60]
		6A-9	Bone	AMS ^14^C	BA160940	42680±300	45790–44680	
Jinsitai (TMP)	45°14’23.4"N, 115°28’32.7"E	7	Bone	AMS ^14^C	BA132054	267540±150	31187–30492	[61]
		7	Bone	AMS ^14^C	UCIAMS180644	34920±360	40840–39390	
		7	Bone	AMS ^14^C	UCIAMS180645	34210±370	40396–38121	
		8	Charcoal	AMS ^14^C	BA121377	25990±110	30691–29995	
		8	Charcoal	AMS ^14^C	BA121378	25640±110	30150–29710	
		8	Bone	AMS ^14^C	Beta439532	39500±420	43866–42432	
		8	Bone	AMS ^14^C	BA132055	35610±230	41216–40255	
		8	Bone	AMS ^14^C	BA132056	34690±270	40495–39329	
		8	Bone	AMS ^14^C	UCIAMS180642	41820±840	46045–43155	
		8	Bone	AMS ^14^C	UCIAMS180643	39320±610	43984–42308	
		8	Bone	AMS ^14^C	BA4480	36285±230	41797–40942	
SDG1	38°30’6.7"N, 106°17’55.2"E	AL	Teeth	U-series	BKY-82042	38000±2000		[63]
(IUP)		AL	Teeth	U-series	BKY-82043	34000±2000		
		AL 6.5m	Charcoal	AMS ^14^C	UGAMS-9682	36200±140	41532–40954	[64]
		AL 6.1m	Quartz	OSL	L1653	43000±3000		[65]
		AL 6.5m	Quartz	OSL	L1654	43000±3000		
		AL 7m	Quartz	OSL	L1655	42000±3000		
		AL 7.6m	Quartz	OSL	L1656	46000±3000		
		AL 7.6m	Quartz	OSL	L2361	35000±3000		
		AL 8.1m	Quartz	OSL	L2362	35000±3000		
		AL 8.6m	Quartz	OSL	L2363	33000±3000		
		AL 9.1m	Quartz	OSL	L2364	33000±2000		
		AL 9.6m	Quartz	OSL	L2365	39000±3000		[66]
		AL 11.5m	Quartz	OSL	IEE1894	34800±1500		
SGD2 (IUP)	38°17’51.8"N, 106°30’9.6"E	AL 6	Quartz	OSL	IEE18895	38300±3500		[66]
		AL 7	Wood	AMS ^14^C	BA7943	36329±215	41800–41000	

The newly excavated Sanlongdong cave site in the Chifeng region of Inner Mongolia represents the easternmost site in northern China in which Mousterian assemblages, including points and Quina scrapers have been found [67]. Detailed information about the lithic assemblage and chronology has not yet been formally reported. However, the excavators argue that the Sanlongdong cave site dated to ~50 ka BP, and the lithic assemblage and tool retouching methods resemble the Mousterian techno-complex more so than the contemporaneous core-flake techno-complex widely distributed in the hinterland of northern China [67].

**Fig 3:**
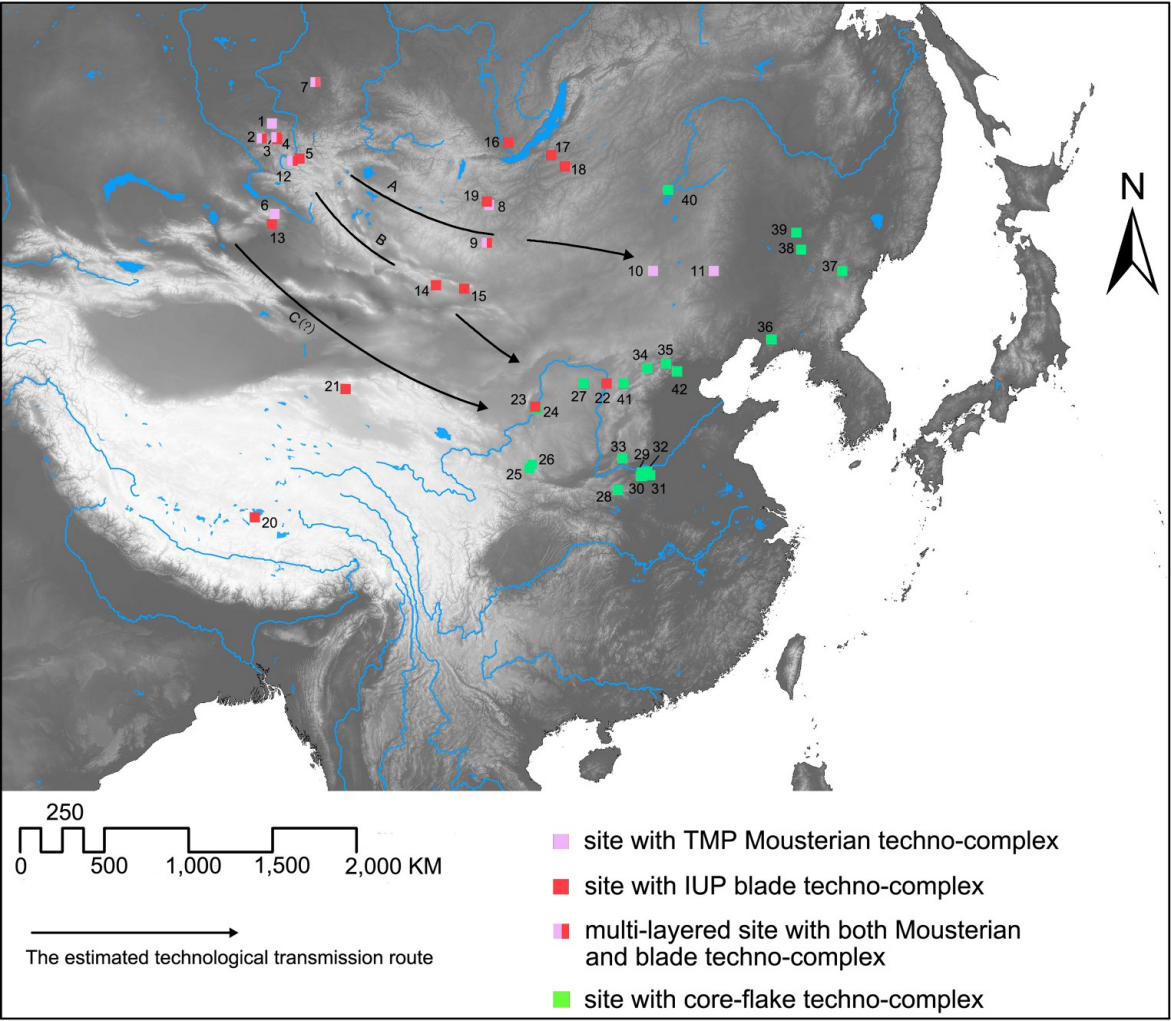
The distributional pattern of the sites with different techno-complexes and the estimated technological transmission route between Mongolia/Siberia and northern China between 50–32 cal ka BP. The base map raster is made from the USGS digital elevation map data from http://earthexplorer.usgs.gov/. (1: Okladnikov cave; 2: Strashnaya cave; 3:Denisova cave; 4: Ust Karakol; 5: Kara-Tenesh; 6: Tongtiandong; 7: Mokhovo-2; 8: Kharganyn Gol-5; 9: Orkhon-1; 10: Jinsitai; 11: Sanlongdong cave; 12: Kara-Bom; 13: Luotuoshi; 14: Chikhen Agui; 15: Tsagaan Agui; 16: Voenny Hospital; 17: Kamenka; 18: Tolbaga; 19: Tolbar-4; 20: Nwya Devu; 21: Lenghu; 22: Yushuwan; 23: Shuidonggou-.1; 24: Shuidonggou-2; 25: Changweigou; 26: ZS08; 27: Wulanmulun; 28: Longquandong; 29: Zhijidong; 30: Fangjiagou; 31: Zhaozhuang; 32: Laonainaimiao; 33: Xiachuan (Fuyuhe); 34: Xibaimaying; 35: Xigouwan; 36: Xiaogushan; 37: Mingyuegou; 38: Zhoujiayoufang; 39: Guxiangtun; 40: Zhalainuoer; 41: Shiyu; 42: Zhoukoudian Upper Cave).

Except for the three cases mentioned above, none of the other Middle Paleolithic sites in China show clear Mousterian features. This general absence of Mousterian assemblages at this time suggests that the Mousterian techno-complex only diffused along a linear stretch on the periphery of northern China, which stretched more than 2000 km from the northwest to northeast (Fig 3). The distribution of sites with evidence of the TMP Mousterian techno-complex across the eastern Eurasian Steppe and northern China (Fig 3) suggests this technology probably diffused into the northern Xinjiang region of Northwest China after passing through the Altai Mountains, and into Northeast China via the central Mongolia Steppe Belt stretching from west to east on the northern edge of the Gobi Desert.

#### The IUP techno-complex in northern China

**Fig 4:**
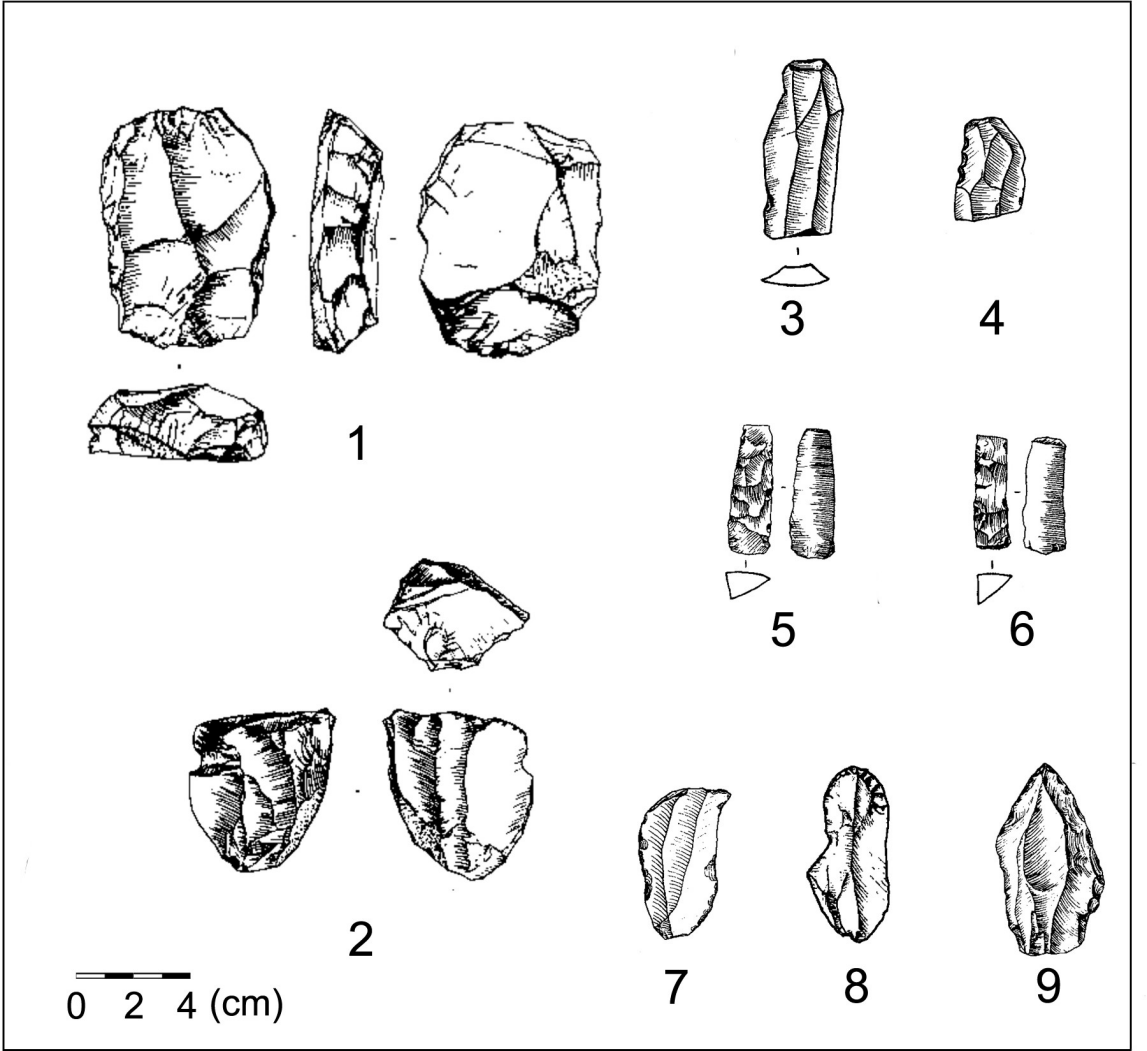
The typical artifacts of IUP techno-complex found in Shuidonggou-1. (1: Levallois blade core; 2: Prismatic blade core; 3: blade (broken); 4: denticulate (broken);, 5,6: crest blade; 7: side scraper; 8: end scraper; 9: Levallois point; 1–9: after [72]).

The IUP techno-complex intruded even further southward than the TMP Mousterian techno-complex. The Shuidonggou site complex located on the margins of the semi-arid Ordos Basin in eastern Northwest China (see #23 and 24 in Fig 3) has been intensively studied and shows evidence of the southeastward diffusion of the IUP techno-complex [68]. The main cultural layer of Shuidonggou-1, Shuidonggou-9, and the lowest cultural layer of Shuidonggou-2 all contain varying amounts of IUP lithic remains [69]. A reexamination of the chronometric data suggests that the IUP techno-complex in the Shuidonggou region dates to 43–33 cal. ka BP [70]. The Shuidonggou site complex is characterized by its strong inheritance of elements of the TMP Mousterian techno-complex. Shuidonggou-1 has the most abundant IUP lithic remains (Fig 4). Levallois flat-faced cores used for blade production far outnumber prismatic/sub-prismatic and narrow-faced blade cores at Shuidonggou-1 [71]. Side scrapers and denticulates are the two most frequently encountered tool types. These side scrapers and denticulates show the inheritance of the Mousterian tradition and form higher proportions of the lithic assemblage than emergent tool types like end scrapers and burins [54]. This pattern suggests that the IUP lithic techno-complex from the Suidonggou site complex more closely resemble that found at the IUP sites in the Siberian Altai and southern-central Mongolia, than at IUP sites in North Mongolia and the Transbaikal region where tools associated with the Mousterian tradition are either absent or only form a low proportion of the total assemblage [16].

Besides the Shuidonggou site complex, a couple of other sites in northern China show strong evidence of IUP techno-complex remains, but most of these artifacts come from surface collections meaning no specific stratigraphic IUP techno-complex contexts were securely dated. The exact number of sites with IUP techno-complex materials remain unclear because some are not reported. Reported sites include the Luotuoshi site complex (see #13 in Fig 3) in the Dzungaria Basin of the Xinjiang Autonomous region [73], and the Yushuwan site (see #22 in Fig 3) in south-central Inner Mongolia [74].

The locations of these sites suggest that the IUP techno-complex was spatially distributed in a linear fashion running west to east across some of the arid and semi-arid basins of Northwest China. Whether or not the IUP techno-complex reached into Northeast China remains unclear since no typical IUP techno-complex remains have been reported here. However, blade assemblages with possible IUP techno-complex influences have been found in the Korean peninsula mixed with lithics belonging to the core-flake techno-complex as early as 40–30 cal ka BP [75]. Since Northeast China is located between Siberia-Mongolia and the Korean Peninsula, it is highly likely that the IUP techno-complex diffused into Korea through Northeast China. This may be further corroborated by the argument that blade technology moved into the Korean Peninsula from the north and west [76]. Our general patchy understanding of the spatial distribution of the IUP lithic techno-complex remains in the region could be remedied by more intensive Paleolithic survey work in Northeast China.

The IUP techno-complex has also been identified at the Lenghu site in the Qaidam Basin on the northern Tibetan Plateau. The Lenghu site was occupied between 37–30 cal ka BP [77] (seen as #21 in Fig 3). This discovery suggests that Northwest China served as a gateway for the technological diffusion of the IUP techno-complex from Siberia-Mongolia onto the Tibetan Plateau. The recent discovery of Nwya Devu further indicates that people occupied the high-altitude areas (above 4500 m) of the Tibetan Plateau at ~40–30 cal ka BP. The lithic assemblage of Nwya Devu resembled contemporaneous lithic techno-complexes found in Siberia and Mongolia because it contained prismatic/sub-prismatic cores which were used to produce large blades. However, Mousterian remains have not yet been identified at Nwya Devu [78].

In comparison to the widespread nature of the IUP on the Tibetan Plateau, the distribution of the IUP techno-complex in northern China was quite limited. Most of North China, Northeast China and the southeastern part of Northwest China were still dominated by the core-flake techno-complex [12]. Although some interregional variations exist in the core-flake techno-complex, there are clear pan-regional similarities which suggest the core-flake techno-complex was a locally inherited tradition which differed dramatically from the TMP and IUP techno-complexes [79]. General similarities in the core-flake techno-complex include the use of irregular small or medium sized flakes as blanks for making tools, the lack of systematic core pre-forming, the light retouch of the tools, and the expedient nature of the toolkits [80]. No obvious geographic barriers or gaps exist between the distributional zones of the IUP and the core-flake techno-complex. In fact, the core-flake techno-complex sites in the Ordos Basin were geographically close to those of the IUP techno-complex in the Shuidonggou area [81]. Moreover, the core-flake techno-complex replaced the IUP techno-complex in the Shuidonggou region at ~33 cal ka BP [82]. Excavations at Shuidonggou-2 and Shuidonggou-7 reveal that core-flake lithic techno-complex layers without any systematic blade and Levallois production were situated stratigraphically above IUP techno-complex layers. Radiocarbon and OSL (Optically Stimulated Luminescence) dating suggest that the main occupational phase associated with the core-flake techno-complex in Shuidonggou-2, Shuidonggou-7, and Shuidonggou-8 occurred ~33–27 cal ka BP [70]. This pattern indicates that the IUP techno-complex was ultimately superseded in the Shuidonggou area by the local core-flake techno-complex.

According to site distributional patterns (Fig 3), the presence of IUP techno-complex materials at the Shuidonggou and Yushuwan sites was probably related to the southeastward technological transmission of this tradition from the eastern Eurasian Steppe. One possible corridor of transmission was through the foothill area of the Gobi-Altai Mountains (marked as A in Fig 3). This foothill area is the nearest path connecting the Altai Mountains and the Ordos Plateau. While this corridor is located in the arid Gobi Desert, the MIS 3 period paleoclimate was more humid than today and sediment studies from paleolakes suggests lake levels were comparatively high [20, 23, 83, 84]. This enlargement of paleolakes is often accompanied with expansion of the surrounding oases as well [85]. Subsequently, it seems likely that the chain of oases presents through the foothill area of the Gobi-Altai Mountains formed a corridor which encouraged movement. As shown in Fig 3 (#14,15), the sites Chikhen Agui and Tsagaan Agui show human activities along the Gobi-Altai Mountains corridor. Another possible corridor of technological transmission runs through the Tianshan-Qilian foothills. This corridor also connects the core area of the eastern Eurasian Steppe with Northwest China. Though most of the Tianshan-Qilian foothill corridor lies in the arid Gobi Desert, again the presence of oases could facilitate movement [86]. Nonetheless, unlike the Gobi-Altai Mountains corridor, little archaeological evidence of IUP techno-complex materials have been identified along the Tianshan-Qilian foothill corridor. However, to date little work has been conducted to identify possible IUP techno-complex materials along this Tianshan-Qilian foothill corridor, meaning any conclusions about which specific route was more important for the introduction of the IUP techno-complex remains somewhat speculative.

### Technological transmission between eastern Eurasian Steppe and the northern China during the MUP period

#### The MUP blade techno-complex in the eastern Eurasian Steppe

Lithic remains attributed to the MUP blade techno-complex are distributed across Mongolia and Siberia from approximately 32–23 cal. ka BP. The MUP techno-complex is characterized by blade and flake reduction technologies from broad-faced and narrow-faced cores. Compared to the IUP techno-complex, the MUP techno-complex shows no evidence of the Levallois technique for blade production, and there is a substantial size variation in both cores and end products [40]. A clear tendency towards microlithization can be detected as bladelets (small blades <12 mm in width) become proportionally more common [87]. These bladelets were often produced from microcores, which were pre-shaped in similar ways to the large blade cores, but are small (usually with height less than 5 cm high). The bladelets and microcores can be found in IUP assemblages, but become far more prevalent in the MUP techno-complex. Side scrapers, end scrapers, burins, gravers, bifaces, and knives were common tool types of the MUP techno-complex [88].

#### The emergence of microblade technology in North China

Our understanding of technological communications between Siberia-Mongolia and northern China in the period between 32–23 cal. ka BP is complicated by the fact that no typical MUP techno-complex assemblages have been identified in northern China. However, a remarkable change in lithic technology happened in China during this period. This change saw microblade technology replace the traditional core-flake technology and become widely distributed across the region. Microblade technology involved the systematic production of tiny, standardized laminar pieces used as insets for composite tools. Usually, these microblades are detached by pressure flaking from specifically shaped pre-formed cores [89, 90]. It is highly debated as to whether microblade technology developed out of the local core-flake techno-complex, or was introduced from Mongolia-Siberia [5, 91–93]. Based on the newest archaeological discoveries, we propose that microblade technology in northern China neither developed *in-situ* from local core-flake technology, nor was imported wholesale directly from Mongolia-Siberia. Instead, we argue it developed out of the previously introduced MUP blade techno-complex from the eastern Eurasian Steppe. Therefore, the emergence of microblade technology in northern China is associated with another wave of technological transmission which differed in nature from earlier transmissions. The specifics of this argument are presented below.

A series of excavations and chronological studies recently reveal that initial microblade technology appeared in North China as early as ~27–26 cal. ka BP [94]. Unlike the later fully-fledged microblade techno-complex, initial microblade technology usually co-existed with blade technology. At some sites, the strata containing mixed microblade and blade assemblages are situated stratigraphically above strata containing purely core-flake techno-complex assemblages [94–97].

**Fig 5:**
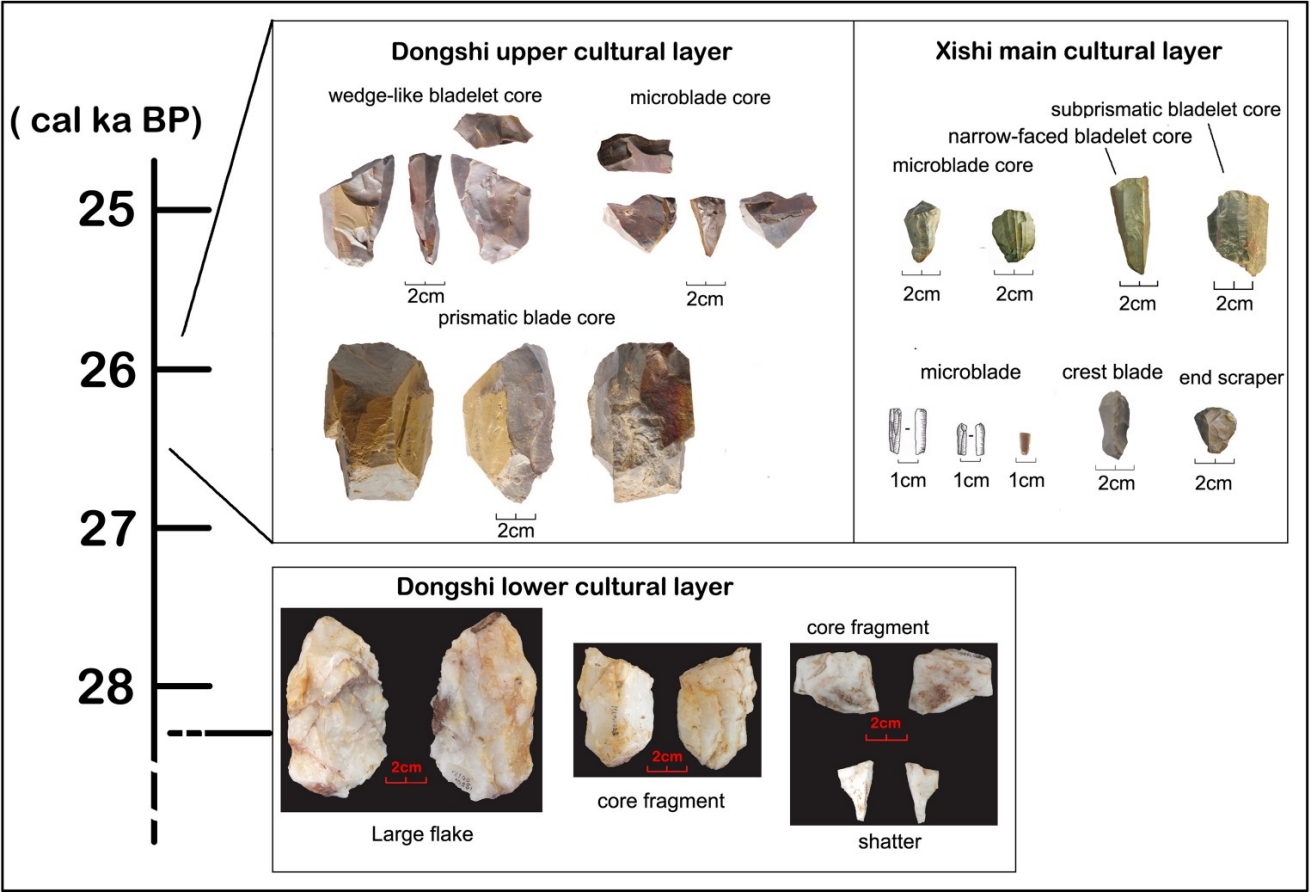
The assemblages associated with the Dongshi lower cultural layer and the Dongshi upper cultural layer/Xishi main cultural layer.

The newly excavated Xishi and Dongshi sites both have mixed microblade/blade techno-complex assemblages. The abundant lithic remains generated from different production stages allows for a systematic chaîne opératoire analysis to compare microblade, blade, and core-flake techno-complex remains to evaluate how comparable they were technologically. The Xishi and Dongshi sites are situated at the transitional zone between the Song Mountain and the Central China Plain in the hinterland of North China. These two sites were open-air lithic workshops for producing blades, bladelets, microblades, and other tools [98, 99]. The microblade/blade layer at the Xishi site was dated at ~26 cal. ka BP [100]. At the Dongshi site, the lower cultural layer with core-flake techno-complex remains is overlaid by an upper cultural layer of microblade/blade remains which is estimated to be contemporaneous with the Microblade/blade layer at the Xishi site (Fig 5) [101]. Radiocarbon samples extracted from an intermediary lens of material between the lower (core-flake techno-complex) and upper (microblade/blade techno-complex) layers of the Dongshi site were dated to 27 cal. ka BP (Fig 5). Despite their stratigraphic proximity to the microblade/blade remains, the core-flake remains in the lower layer of the Dongshi site do not show any transitional features between typical core-flake and microblade technology (Fig 5). Instead the core-flake techno-complex remains are similar to the core-flake remains distributed in surrounding regions from the beginning of the Upper Paleolithic. Similarities between these materials include a reliance on quartz as a raw material, the production of irregular flakes without intensive retouch, and the lack of formalized or standardized end products [101, 102]. Similar patterns detected at other contemporaneous multi-layered sites, such as Shizitan-29, Xiachuan, and Xishahe, suggest pronounced technical differences between core-flake remains and overlaying blade/microblade remains [95–97]. This abrupt technological shift from core-flake to microblade production without a clear transitional techno-complex in between suggests it is unlikely that microblade technology emerged out of the local core-flake technology.

**Fig 6:**
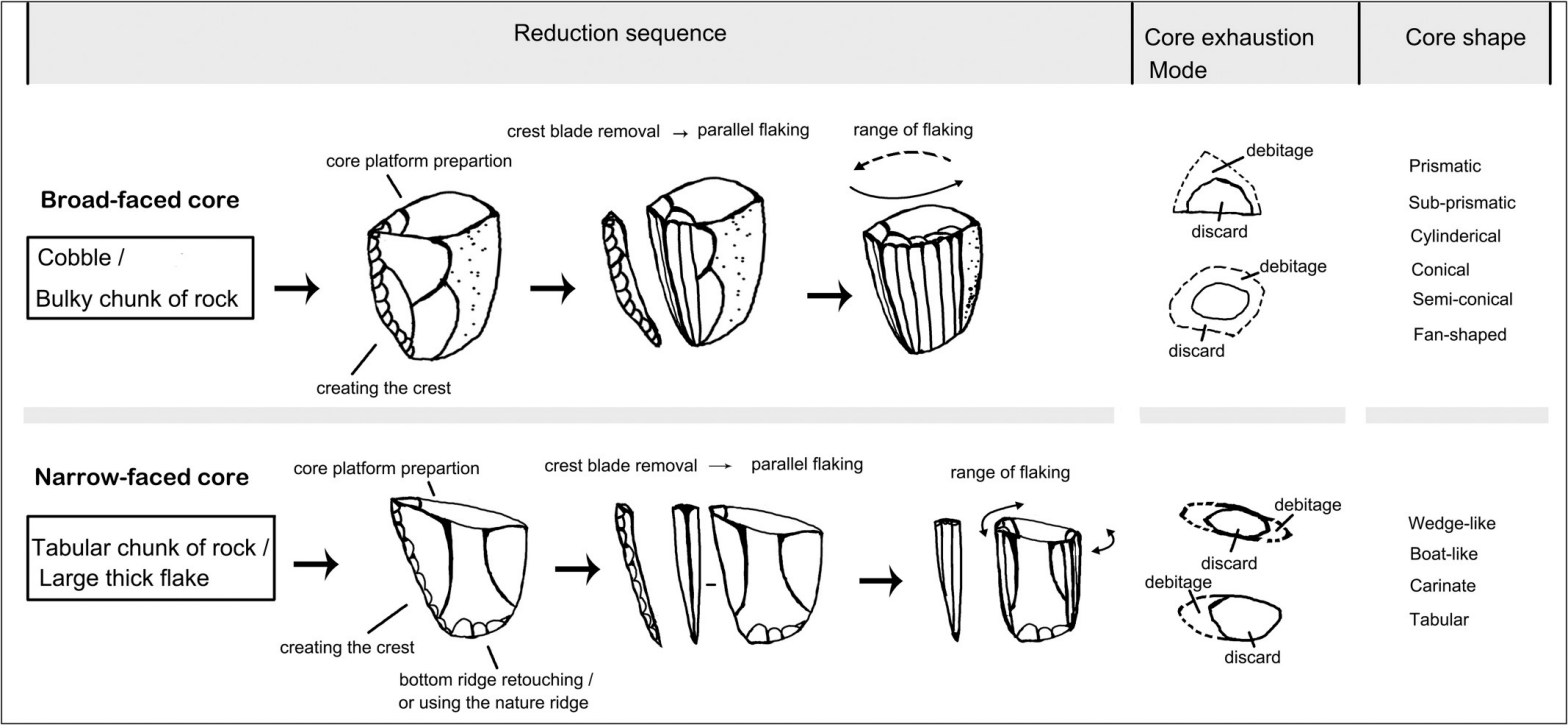
Illustration showing the reductional sequence of broad-faced core production and narrow-faced core production.

In contrast, a chaîne opératoire analysis of the lithic debitage generated during different production stages at the Xishi and Dongshi sites suggests that the microblade remains show strong technical affinity with coexisting blade technology. Both blade and microblade technology are produced using similar technical procedures. Similarities include core pre-shaping, striking platform trimming, and debitage surface maintenance to achieve the persistent parallel flaking of regularized end products [99]. Two different blade core pre-forming and reduction strategies, namely broad-faced and narrow-faced core production was also applied to microblade production (Fig 6). Nonetheless, the thinner and more standard form of the microblade, as well as some specific features along the dorsal and ventral surface of microblades (like the maximum width at the shoulder below the bulb, the small point-like pressure bulb, straight profile, and the curved distal section ending in “feathering”) indicate that unlike blades or bladelets which were often produced by soft hammer percussion, the microblades were produced using pressure flaking [103]. Therefore, early microblade technology mainly differs from blade technology in the use of more diminutive cores, and the use of pressure flaking to produce smaller and more regular end products. Despite these differences, both the microblade and blade production techniques share technical similarities. For this reason, we argue that it was highly likely that the microblade technology which emerged in northern China was a technical innovation based upon existing blade technology rather than a new technological invention.

The blade remains which coexisted with microblade remains at the Xishi and Dongshi sites are similar to contemporaneous MUP blade techno-complex materials present at sites in Siberia and Mongolia. Similarities between the Xishi and Dongshi sites and those in Siberia and Mongolia include: (1) a wide range of size variation in cores and end products (with large blades longer than 10 cm and bladelets as small as 2–3 cm in length, and less than 1 cm in width); (2) bladelets proportionally outnumber large blades (forming the predominant end products); (3) the use of both broad-faced and narrow-faced cores for producing blades but not Levallois cores [99].

**Fig 7:**
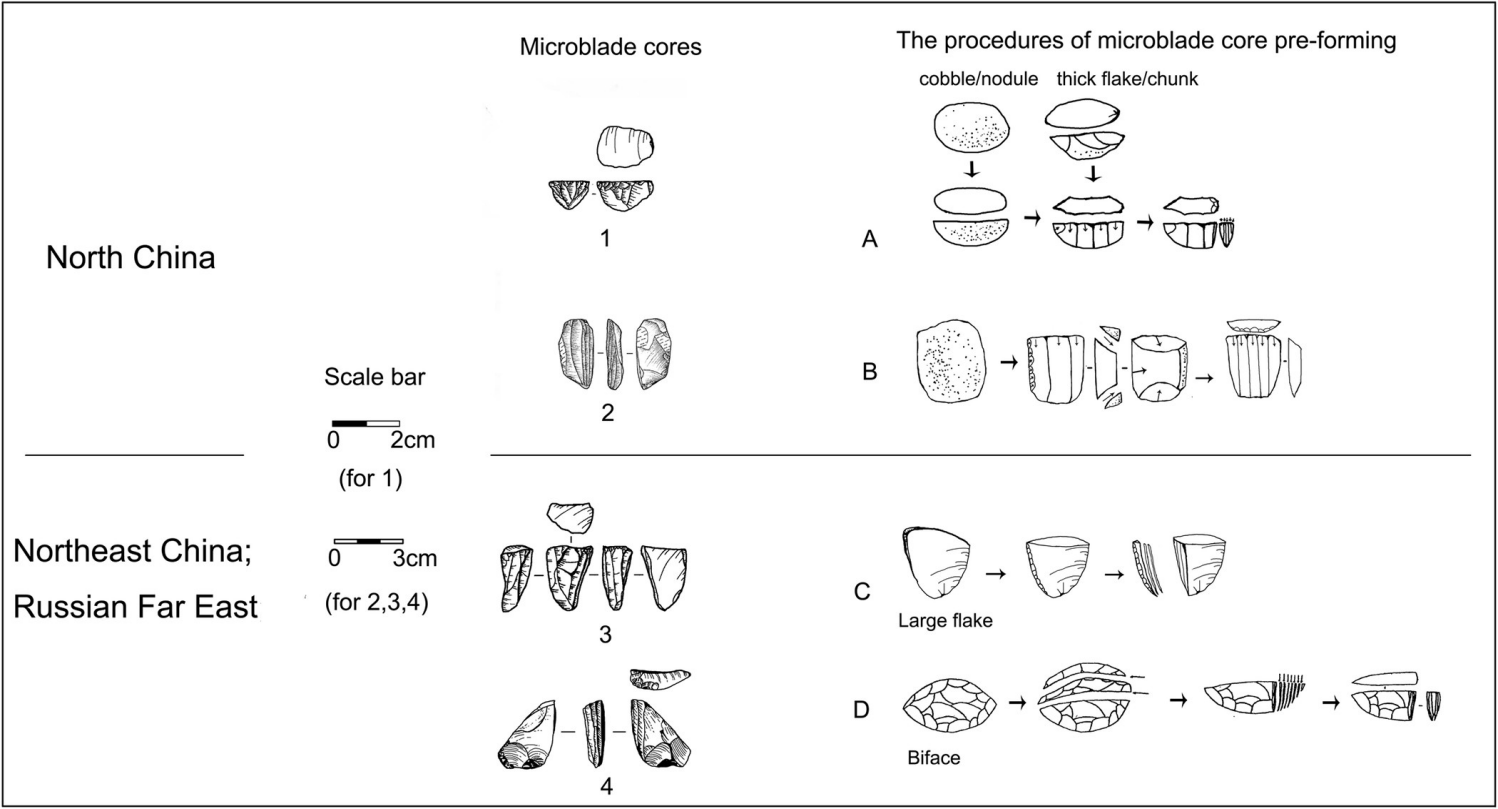
A comparison of the microblade core pre-forming methods between North China and Northeast Asia (include Northeast China, Russian Far East, and Korean Peninsula (1: Boat-shaped core from Longwangchan; 2: Semi-conical core from Shizitan; 3: Wedge-like core from Xishantou; 4: Wedge-like core with Yubetsu method from Ust-Ulma; 1: After [112]; 2: After [108]; 3: After [111]; 4: After [113]).

Chronometrical studies show that stratigraphic layers containing an admixture of microblade and blade assemblages in North China date to 27–25 cal ka BP (Table 2). The proportions of microblades in lithic assemblages increase over time. Core pre-forming techniques developed with increasingly fixed procedures based upon using smaller nodules, resulting in the prevalence of smaller and more standardized microblade cores. These tendencies in core-preforming are evident at different sublayers of Shizitan-29, Longwangchan, and Xiachuan [104–106]. Several core forms develop which seem to be derived from either the broad-faced or narrow-faced cores. These new core forms include the semi-conical and boat-shaped cores (Fig 7: 1–2 and A, B). Semi-conical and boat-shaped cores were the most popular core types between 26–20 cal. ka BP [105, 107]. With the development of microblade technology, blade technology became rarer and ultimately disappeared from lithic assemblages after 25–24 cal. ka BP [108]. This trajectory suggests that microblade technology in North China was developed based upon modification of the introduced MUP blade technology from Mongolia and Siberia. This emergent microblade technology then replaced blade technology as it developed.

**Table 2:** Chronological information about the site stratum with coexisted blade and microblade remains. The radiocarbon data was calibrated by using OxCal 4.4 and IntCal20 calibration curve.

Site	Location	Layer/	Dated	Dating	Lab	Date		Reference
		Context	Material	Method	No.	Uncal. BP	Cal. BP	
Xishi	34°26’38.82"N, 113°13’20.16"E	②c	Charcoal	AMS ^14^C	BA10891	22090±90	26574–26045	[99]
			Charcoal	AMS ^14^C	BA10892	21880±80	26309–25899	
			Charcoal	AMS ^14^C	BA10893	22010±90	26490–25986	
Dongshi	34°26’43.5"N; 113°13’38.16"E	②B	Charcoal	AMS ^14^C	BA142175	23040±80	27551–27154	[99]
			Charcoal	AMS ^14^C	BA142176	23350±80	27741–27395	
			Charcoal	AMS ^14^C	BA142178	24160±100	28510–27891	
Shizitan	36°02’54"N, 110°35’22"E	7	Bone	AMS ^14^C	BA121960	21690±80	26230–25800	[95]
S29			Bone	AMS ^14^C	BA101445	20510±90	24990–24309	
			Charcoal	AMS ^14^C	BA101442	20010±70	24212–23360	
			Bone	AMS ^14^C	BA101439	19650±80	23842 = 23360	
Longwang-	36°09’45"N, 110°26’15"E	5	Charcoal	AMS ^14^C	BA06008	21920±80	26370–25945	[106]
Chan			Charcoal	AMS ^14^C	BA06007	21740±115	26317–25821	
			Charcoal	AMS ^14^C	BA091132	22105±50	26715–25995	
			Charcoal	AMS ^14^C	BA091133	22200±75	26894–26965	
Xishahe	39°55’15.8"N, 114°47’6.2"E	3A	Charcoal	AMS ^14^C	471.485	22680±80	27251–26480	[97]
		3A	Bone	AMS ^14^C	397.247	22800±90	27314–26965	
		3A	Bone	AMS ^14^C	397.241	22690±90	27260–26481	
		3A	Bone	AMS ^14^C	377,941	23070±90	27611–27209	
Xiachuan	35°26’32"N, 112°01’17"E	IT2-6	Charcoal	Conv ^14^C	ZK-0384	21086±1000	27602–23234	[109]
		IT8(4)	Charcoal	Conv ^14^C	ZK-0417	23224±1000	29914–25813	
		?	Charcoal	Conv ^14^C	ZK-0393	20115±600	25694–22993	
		2-3A	Charcoal	AMS ^14^C	BETA-	n.a.	~27000–25000	[96]
Youfang	40°14’N, 110°41‴E	Layer 2.1m	Quartz	OSL	L2304	26600±2100	-	[110]
		Layer 2.5m	Quartz	OSL	L2305	25100±2000	-	
		Layer 2.9m	Quartz	OSL	L2306	29200±2000	-	
Xishantou	46°43’46.25"N, 123°00’40.32"E	3	Bone	AMS ^14^C	BETA-	23680±170	28296–27432	[111]
		3	Bone	AMS ^14^C	BETA-	23610±80	27895–27648	

#### Emergence of microblade technology in Northeast China

The lithic assemblage of the Xishantou site in the western part of the Northeast China Plain shows similarities with the assemblages of the Xishi and Dongshi sites. Bladelet production and the incorporation of early-stage microblades as subordinate end products predominate in the Xishantou site lithic assemblage [111]. In contrast to the Xishi and Dongshi sites, the Xishantou site only has narrow-faced blade and microblade cores in tabular or wedge-like shapes, and there are no typical broad-faced prismatic/sub-prismatic cores [111]. Furthermore, the mixed blade/microblade layer at the Xishantou site dated to as early as 28 cal ka BP, which is roughly 1–2 ka BP earlier than the blade/microblade layers at the Xishi and Dongshi sites.

Another site with blade remains is the Shibanzhan site in the Greater Khingan Mountainous region. A tabular-shaped blade core with bidirectional flaking along the narrow face was found, accompanied with four blades. OSL dating indicates that this cultural layer dated to ~25 ka BP [114]. No microblade remains have been found at the Shibanzhan site. However, since only 24 lithic pieces were recovered from excavation, it remains unclear whether the absence of microblade remains is due to sampling bias.

Around 27–23 cal. ka BP, microblade remains became more widely distributed in Northeast China and its eastern surroundings. This growing prevalence of microblade remains is indicated by the dating of the earliest microblade assemblage layers at sites like Dadong in the Changbai Mountainous Area of Northeast China, Ust-Ulma in the Russian Far East, Kashiwadai-1 and Pirika-1 in Hokkaido, Hopyeond-dong and Sinbuk on the Korean Peninsula, and Ogonki-5 on Sakhalin Island [115–118]. Unlike contemporaneous early microblade technology in North China, the microblade cores in these regions were dominated by a specific type of narrow-faced core which is often called the Yubetsu core in the East Asian archaeological literature [119]. Yubetsu cores were initially formed as a small biface. The lateral edge of the biface blank was then removed to create a narrow deck-like platform. Multiple spalls were subsequently removed along the narrow end of the platform to create a narrow-faced debitage surface for the consecutive production of microblades by pressure flaking (Fig 7: D) [107]. Production using a Yubetsu core reflects a combination of blade and bifacial techniques for making microblades.

We argue that the compilation of this evidence suggests that microblade technology in Northeast China emerged from the narrow-faced blade core production strategy of the MUP techno-complex. The initial microblade technology differs from blade technology mainly because smaller cores and perhaps pressure flaking methods were used. Later in time, innovative methods were applied to core pre-forming. As a result, overall core shape still resembled narrow-faced cores, but the whole reductional sequence from core pre-forming to consecutive microblade production differs remarkably from any of the blade production methods.

#### MUP technological diffusion and the emergence of microblade technology in northern China

According to the analysis and overview above, the earliest microblade remains often coexisted with blades and show strong technical affinity with them. Subsequently, microblade technology became further developed with a higher level of stylization of core pre-forming. The specific methods of microblade core-preforming in North China remain very similar to the core-preforming techniques employed for blade production. However, in Northeast China and its eastern surroundings more innovative types of core-preforming developed. These novel core-preforming techniques resulted in greater differences between blade and microblade traditions in Northeast China than in North China. Hence, microblade technology in northern China can be considered a localized innovation based upon blade technology. Different trajectories of localized innovation can be found across North China and Northeast China, leading to the formation of different types of microblade technology.

As discussed above, the microblade technology was likely based upon the introduced MUP techno-complex. Two additional lines of evidence support this inference and exclude the possibility that microblade technology emerged out of the earlier IUP techno-complex. Firstly, the IUP techno-complex only appeared ephemerally in a spatially restricted fashion in specific regions of northern China and the IUP techno-complex materials in some of these contexts was superseded by the core-flake techno-complex before 30 cal ka BP, leaving a temporal gap between the IUP and the microblade/blade techno-complexes [13]. Secondly, the blade products found alongside incipient microblade remains show clear features of microlithization, which differs from the large IUP blade remains but is similar to the MUP blade assemblage. Therefore, it seems probable that microblade technology originated from a separate episode of lithic technological diffusion from the eastern Eurasian Steppe to northern China at ~28–25 cal ka BP. Yet unlike the earlier TMP/IUP technological diffusion which involved the wholesale introduction of a complete suite of original IUP techno-complex forms, MUP technology underwent local modification and adaption which ultimately resulted in the appearance of the microblade technology. The widespread distribution of early microblade technology implies that this episode of technological diffusion penetrated much further into the hinterland of northern China and impacted broader regions of northern China than the earlier waves of TMP/IUP technological diffusion.

**Fig 8:**
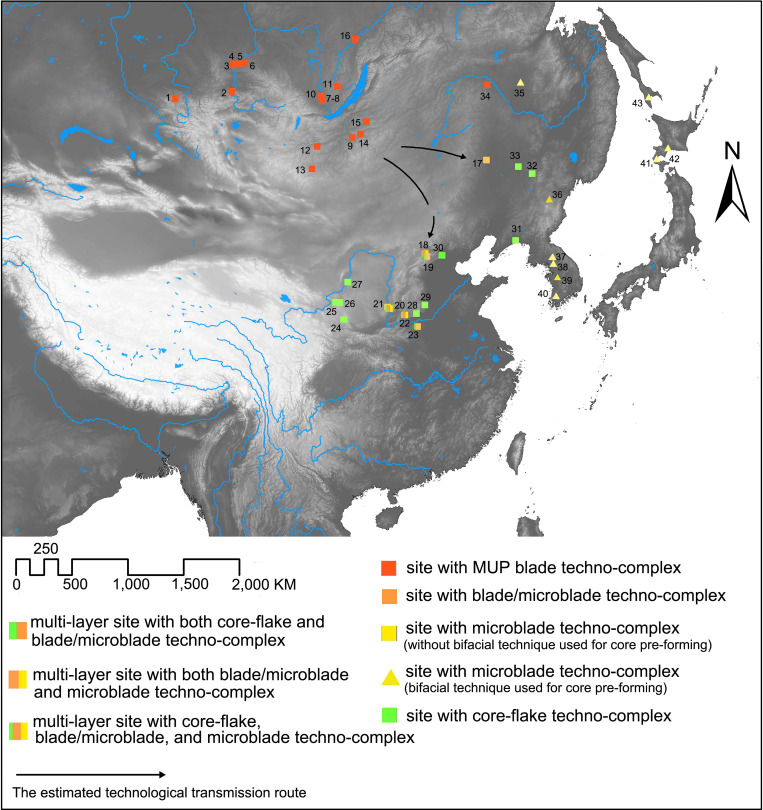
The distributional pattern of the sites with different techno-complexes and the estimated technological transmission route between northern China and Mongolia/Siberia during 32–23 cal ka BP. The base map raster is made from the USGS digital elevation map data from http://earthexplorer.usgs.gov/. (1: Anui-2; 2: Ui-l; 3: Sabanikha; 4: Novoselovo-13; 5: Kurtak-4; 6: Derbina-5; 7: Malta; 8: Buret’; 9: Chitkan; 10: Krasny Yar-1; 11: Shishkino-8; 12: Dorolj-1; 13:Orkhon-7; 14: Priiskovoe; 15: Tolbaga; 16: Alekseevsk 1; 17: Xishantou; 18: Youfang; 19: Xishahe; 20: Shizitan-2929; 21: Longwangchan; 22: Xiachuan; 23: Xishi/Dongshi; 24: ZL05; 25: TX08; 26: TX03; 27: Shuidonggou-2; 28: Tashuihe; 29: Xiaonanhai; 30: Dongfangguangchang; 31: Miaohoushan; 32: Xuetian; 33: Yanjiagang; 34: Shibazhan; 35: Ust’-Ulma; 36: Dadong; 37: Jangheung-ri; 38: Hopyeong-dong; 39: Daejeong-dong; 40: Sinbuk; 41: Pirika-1; 42: Kashiwadai-1; 43: Ogonki-5).

Fig 8 shows that the earliest sites with MUP blade techno-complex influences (apparent in blade and microblade mixed techno-complex assemblages) were found in North and Northeast China, not Northwest China. Sites in Northwest China during 28–23 cal. ka BP still had core-flake techno-complex assemblages and the transition to microblade technology in this region occurred later than that in North and Northeast China [120]. This pattern reveals that the regions impacted by the MUP techno-complex were concentrated in the eastern part of northern China and not in Northwest China. It further indicates that unlike the earlier southeastward TMP and IUP technological transmission through Northwest China, the MUP blade techno-complex was probably transmitted into northern China through the corridors connecting the eastern part of the eastern Eurasian Steppe with northern China. The specific routes likely involved diffusion from Mongolia and South Siberia southward along the eastern margins of the Gobi Steppe into North China, and eastward past the gateways of the Great Khingan Mountains into Northeast China (Fig 8).

### The technological transmission between northern China and the eastern Eurasian Steppe during the Late Upper Paleolithic

#### The divergent development of microblade technology between the north and south of northern China

**Fig 9:**
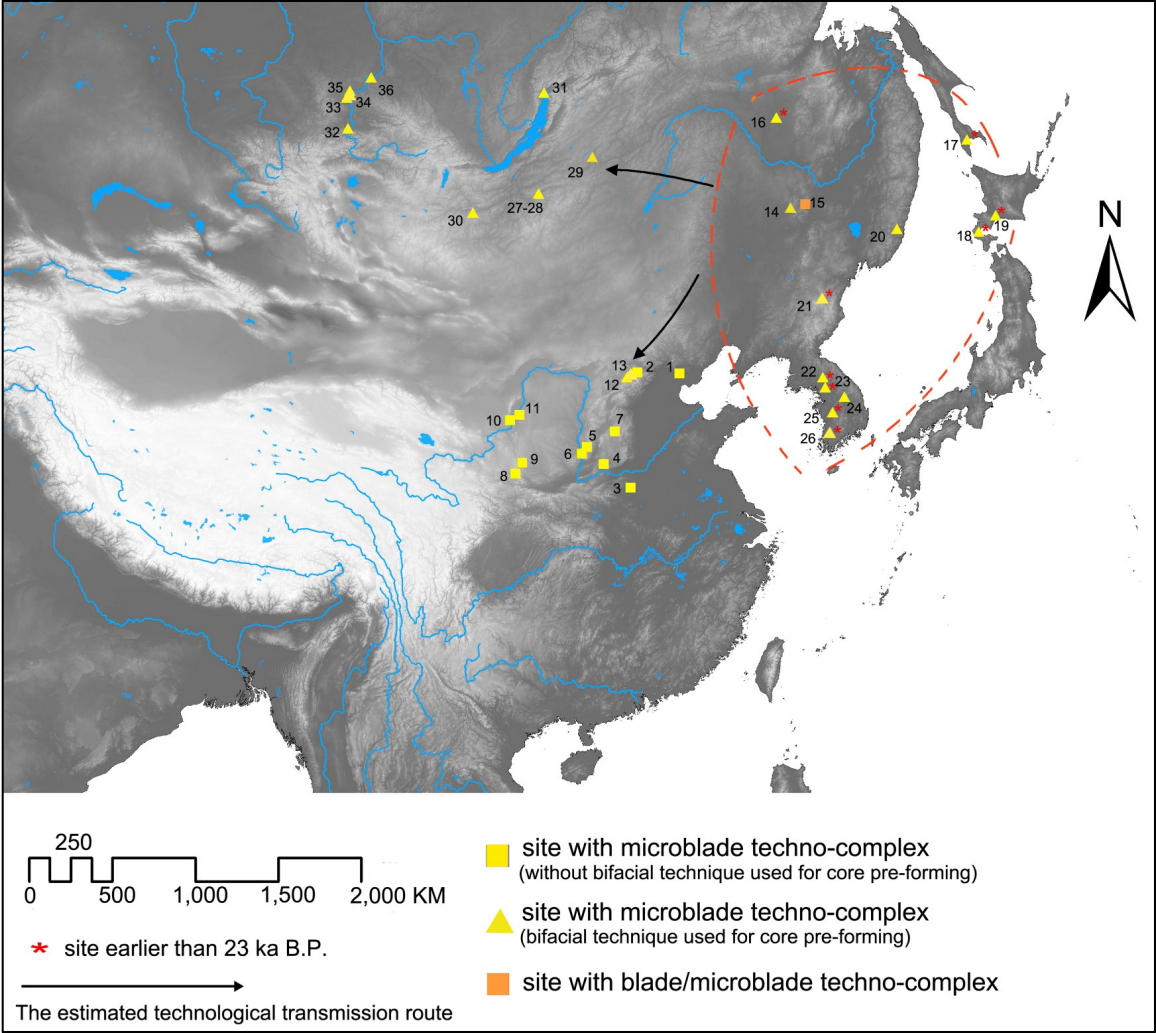
The distribution of sites with different techno-complexes and the estimated technological transmission route between northern China and Mongolia/Siberia during 23–11 cal ka BP. The base map raster is made from the USGS digital elevation map data from http://earthexplorer.usgs.gov/. (1: Mengjiaquan; 2: Erdaoliang; 3: Lingjing; 4: Xiachuan; 5: Xueguan; 6: Shizitan-1; 7: Mengjiazhuang; 8: Shixiakou; 9: PY-03; 10: Pigeon Mtn.; 11: Shuidonggou-12; 12: Hutouliang; 13: Maanshan; 14: Taoshan; 15: Huayang; 16: Ust’-Ulma; 17: Ogonki-5; 18: Pirika-1; 19: Kashiwadai-1; 20: Suvorovo-4; 21: Dadong; 22: Jangheung-ri; 23: Hopyong-dong; 24: Suyanggae; 25: Daejeong-dong; 26: Sinbuk 27: Ust-Menza-2; 28: Studenoe-2; 29: Sokhatino-4; 30: Tolbar-15; 31: Kurla-3; 32: Oznachennoye-1; 33: Tashtyik-4; 34: Kokorevo; 35: Novoselovo-7; 36: Afontova Gora).

Microblade technology is characterized as being prevalent across northern China and Mongolia-Siberia during the LUP period (Fig 9) [121]. Two types of the microblade techno-complex can be identified based mainly on divergent core-preforming and reduction trajectories. The microblade techno-complex in North China and Northwest China mainly involved diverse core forms for producing microblades, including broad-faced cores of conical, semi-conical, and cylindrical shapes, and narrow-faced cores like the wide wedge-shaped, or boat-shaped cores. No bifacial techniques were applied for core pre-forming [107, 122]. In contrast, the microblade techno-complex in Northeast Asia, the Russian Far East, the Korean Peninsula, Hokkaido, Northern Honshu, Mongolia, and Siberia showed a predominance of narrow wedge-like cores used for microblade production [123]. Bifaces or flakes were frequently used as blanks for cores and various techniques were applied for creating and maintaining striking platforms and debitage surfaces of bifacially formed cores [88, 122, 124, 125].

#### The expansion of narrow wedge-like microblade core technology into Siberia, Mongolia, and North China

The development of two divergent reductional trajectories of microblade production are apparent in North and Northeast China at 28–23 cal. ka BP (as discussed above). Yet during the time period between ~23–11 cal ka BP, a microblade techno-complex with a predominant reliance on narrow wedge-like cores for production expanded into vast areas of Siberia and Mongolia (Fig 9). The timing of the emergence of microblade technology in Siberia is highly debated. Some scholars argue that IUP and MUP techno-complex microcores are prototypes of these later microblade cores, and that the microblade technology developed *in situ* from the IUP and MUP techno-complex [90, 126, 127]. Other scholars note that this new Siberian microblade techno-complex comprises many features of microblade assemblages elsewhere such as the aforementioned narrow wedge-like cores, use of pressure flaking, and highly standardized tiny laminar end products. These scholars draw upon these similarities and emphasize the discontinuity between microblade technology and the MUP blade techno-complex to argue that the appearance of microblades in Siberia resulted from population dispersal from the east [88, 128–130]. The focus of this debate mainly rests on how we define microblade technology. If we fastidiously define microblade technology as the systematic use of pressure flaking to produce highly standardized laminar end products from specific pre-formed cores, then unequivocal evidence of microblade technology in Siberia did appear later than in North China and Northeast Asia (including Northeast China, the Russian Far East, Hokkaido, and the Korean Peninsula) [103]. The sites which show the earliest remains of microblade technology in South Siberia include the Studenoe-2 and Ust-Menza-2 found in the Transbaikal region, which date to 23–22 cal. ka BP; almost 4000 years later than its appearance in Northeast China and its eastern surroundings [130, 131].

Due to the rarity of sites with clear stratigraphy, it is difficult to ascertain when the oldest microblade technology appeared in Mongolia. Nonetheless, unequivocal microblade remains coming from the definite cultural layers was found in the horizon 3 and 4 of the Tolbar-15 site, which is dated to around 18–17 cal. ka BP. Like those in Siberia, these microblades were fashioned from narrow wedge-like cores [132].

Outside of Mongolia and Siberia, the expansion of microblade technology centered on the use of narrow wedge-like cores (the Yubetsu technique) is also identified in the Nihewan Basin of northern North China (Fig 9). A number of sites dating to ~17–15 cal. ka BP show the exclusive use of narrow wedge-like cores for microblade production, forming a direct contrast to the earlier boat-shaped cores which were used in this region [107, 133]. The spread of narrow wedge-like microblade cores is constrained to a limited region in the northern part of North China, this stands in stark contrast to the ubiquity of these cores across vast areas of Northeast China, Mongolia, and Siberia [134].

## Discussion

**Fig 10:**
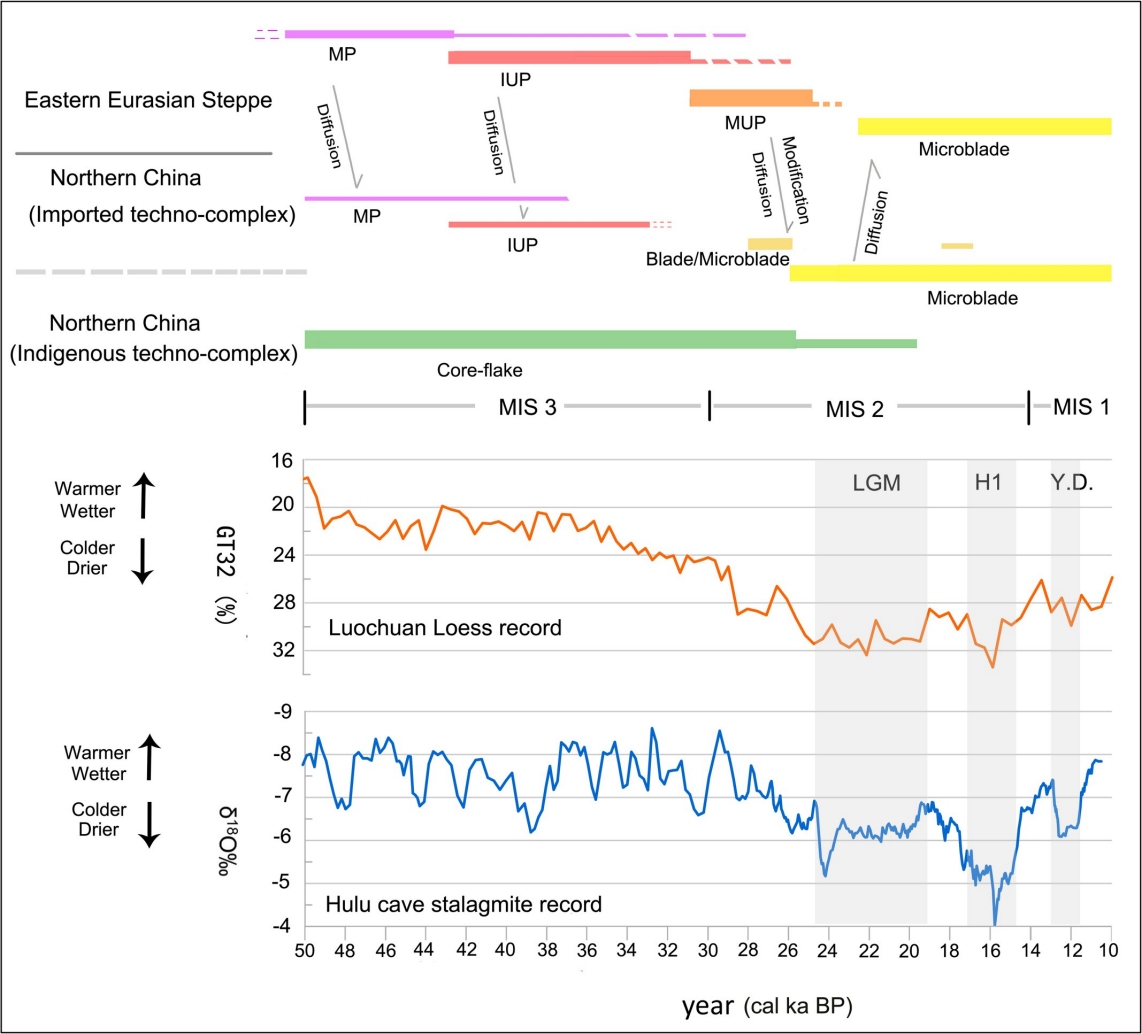
Temporal correspondences between cultural changes and climatic fluctuations in northern China and the eastern Eurasian Steppe (Mongolia, South Siberia) during 50–11 cal. ka BP. The paleoclimate curve was made based on the data from [135] and [136].

Multiple episodes of technological transmission can be identified from the distributional patterns of lithic techno-complexes, indicating consecutive cultural communication between the eastern Eurasian Steppe and northern China from the TMP to the end of the Upper Paleolithic. Each episode shows distinct patterns of technological transmission as summarized in Table 3 and Fig 10.

**Table 3:** Summary of the patterns of each wave of technological transmission between the east Eurasian Steppe and northern China in the Late Pleistocene.

Dates (cal ka BP)	Technology (transmitted)	Transmission Direction	Source of Transmission	Target of Transmission	Environmental Background
~50–38	TMP Mousterian	Northwest to southeast	Siberia, Mongolia	Periphery of Northwest and Northeast China	MIS 3 (warmer/wetter; high paleolake level period)
~43–33	IUP blade mixed with Mousterian	Northwest to southeast	Siberia, Mongolia	Parts of west and east of Northwest China; Tibetan Plateau, parts of Northeast China (probably)	MIS 3 (warmer/wetter; high paleolake level period)
~28–25	MUP microlithized blade	North to south and east	Siberia, Mongolia	Widespread across North China, Northeast China	MIS 2 (colder/drier, right before LGM)
~23–20	LUP microblade	East to west, north, and southwest	Northeast China and its eastern surroundings	Siberia, Mongolia, Northern periphery of North China	MIS 2 (colder/drier, after the peak of LGM)

Table 3 and Fig 10 show that the technological transmission of lithic techno-complexes between the eastern Eurasian Steppe and northern China happened at both warmer/wetter and colder/drier periods. Technological transmission moved along different pathways at different times. These different waves of transmission had different impacts; some culminated in widespread distribution of new technologies and profound changes in local technological development while others resulted in relatively restricted distributions with no obvious alteration of local technology. The reason why each episode of technological transmission differed in this respect probably rested on the nature of lithic technology and the extent to which it was suitable for local environmental conditions.

### Technological organization, adaptive strategy, and cross-regional diffusion

The TMP and IUP lithic techno-complexes, which are characterized by the prevalence of Mousterian or blade and Mousterian mixed techno-complex assemblages, only spread into the north and west peripheries of northern China and did not penetrate into North China. The indigenous core-flake techno-complex still thrived in most parts of northern China. There was no pronounced geographical distance between the distributional area of the core-flake techno-complex and the TMP and IUP techno-complexes (Fig 3). Moreover, at about 30–25 cal. ka BP, the core-flake technology even replaced the IUP techno-complex at Shuidonggou area. Such patterns imply that the TMP and IUP techno-complexes which were introduced during the MIS 3 period only had limited impact on the development of local lithic technology in northern China. The restricted distribution and limited impacts of these imported techno-complexes in northern China probably resulted from the fact that they are more sophisticated lithic techno-complexes which have higher production costs, but did not pose sufficient advantages to outcompete the simple local technologies at this time.

The TMP and IUP lithic techno-complexes involve the use of large and fine-grained raw materials, complicated core pre-forming procedures, sequential flaking of regular pieces of end products and intensive retouch of tools. They not only demand higher lithic reduction skills but also require a more restricted array of raw materials [55, 137]. The common type of lithic raw materials used for core-flake production in northern China, such as quartz and quartzite, are unsuitable for use as Levallois or large blade cores. Hence, people had to search for fine-grain cryptocrystalline (like cherts) if they adopted the TMP or IUP techno-complexes. However, northern China has limited cryptocrystalline resources outside some constrained regions of Northwest and Northeast China [138]. The quartz and quartzite in many regions (especially North China) are more easily obtainable than cryptocrystalline [138]. Although the availability of raw materials did not directly determine the prevalence of lithic techno-complexes, the limited distribution of the necessary fine-grained raw materials would obviously increase raw material search times in many parts of northern China if people adopted the TMP or IUP lithic techno-complex.

Therefore, it is reasonable to assume that unless Mousterian and blade technology provided urgently needed benefits which could not be offered by the core-flake technology, they would not be widely adopted in northern China. Compared to core-flake technology which involves mostly expedient tools, one benefit of Mousterian and blade technology was the creation of formalized end products with pre-planned forms [55, 139]. The formality and refined retouch could maximize the efficiency and the use life of the tools [10, 140, 141]. Hence, these tools are most suitable to conditions in which there is a limited temporal window for people to efficiently acquire resources without interruptions associated with repairing and retooling [142]. These circumstances might involve situations in which people are highly reliant on seasonal access to mobile animal resources in specific locales. The failure to acquire such resources could be catastrophic if few other stable resources were available [142]. Generally, such situations occur in higher latitude areas, or in either arid or high elevation low latitude areas where the available food resources are extremely seasonal and there are fewer edible plants to exploit [143].

While more detailed paleoenvironmental reconstruction is needed, the current paleoenvironmental research implies that broad regions of northern China, especially its southern part, did not conform to the environmental conditions mentioned above during the relatively warmer and wetter MIS 3 period [12, 24, 33, 144]. From a broad geographical perspective, the southern and eastern parts of northern China are similar to the humid low-latitude areas with sub-tropical climatic features, whereas its northern and western parts closely resemble the semi-arid high latitude regions with cool temperatures [79]. During warmer environmental periods like MIS 3, people in the eastern and southern parts of northern China had access to a diverse array of resources and could depend on edible plants using expedient toolkits [12]. These circumstances meant that a techno-complex geared towards quickly acquiring time sensitive resources was not urgently needed, and the simple core-flake technology was viable for undertaking subsistence activities [145]. The logic of this argument explains why the core-flake techno-complex prevailed across broad regions of northern China, while the TMP/IUP techno-complexes were mainly confined to the northernmost parts of northern China. The reason why the spread of the TMP/IUP techno-complexes was limited in the northernmost part of northern China is because this region was not only spatially closer to the Eurasian Steppe, but was also more ecologically similar to it than the south part of northern China.

In contrast to the episode of transmission associated with the TMP/IUP techno-complex during the relatively warm and humid MIS 3 period, the MUP blade technology diffused into northern China just before the LGM when the climate was colder and drier (Fig 10). This episode of transmission impacted a much broader area of northern China, but probably occurred through local modification of newly introduced technology rather than the direct wholesale adoption of it. Two distinct phenomena are highlighted to better understand this episode of transmission: (1) according to current data, MUP blade remains always coexist with early forms of microblade remains across northern China; (2) blade remains disappeared from the lithic assemblages once microblade technology became sufficiently developed (~25–24 cal. ka BP). This indicates that modification of the introduced MUP blade technology happened soon after its transmission into northern China. The microblade technology which evolved from the modification offered adaptive advantages for local environmental conditions and therefore thrived and replaced blade technology.

Microblade technology was more suitable for the local environment in northern China because it lowered the cost of raw material acquisition and better allowed people to cope with the climatic changes associated with the LGM period. Although Microblade technology also involved sophisticated flaking procedures and high demands for quality raw materials, the use of small cores to produce extremely small end products enabled people to use just tiny nodules or flakes of quality raw materials. Therefore, although quality raw materials were only scarcely distributed in most parts of northern China, they could be fully exploited in the most economical way.

Moreover, from the perspective of technological organization, microblade technology provided adaptational benefits for dealing with environmental changes during the LGM period. Firstly, microblades were often used as insets slotted into different organic handles or shafts to form composite tools for different purposes (such as points for hunting, or knives for butchering) [146]. The standardization of microblade shapes make these composite tools easy to maintain because broken or lost insets could be easily replaced with spare insets of similar sizes [18]. This formalization of microblade technology increased the flexibility of the tools and reduced repair and retooling time. The reduction in repair and retooling times ensured uninterrupted tool use during subsistence activities. Also, both microblade cores and microblades were light-weight and easily portable [147]. Therefore, microblade technology has better performance or efficient and intensive exploitation of seasonal resources [148].

The shift to a drier and colder environment right before and during the LGM period caused the southward movement of ecological zones. The retreat of forests and the expansion of desert-steppe, steppe, and meadow-steppe across northern China, made large portions of the region more ecologically similar to the Eurasian Steppe [32]. This situation made the benefits of microblade technology appealing because it better allowed people to adjust to these new environmental conditions. The shift to a drier and colder environment would have necessitated tools which allowed people to fully tap into time-sensitive resources to cope with shrinking growing seasons, decreased resource diversity and richness, as well as resource fluctuations due to a drier and colder climate.

Furthermore, the performance of microblade technology in coping with resource stress might explain why it then diffused into Siberia and Mongolia after the peak of the LGM. The scarcity of sites and chronological records between 25–23 cal. ka BP implies that the higher latitude eastern Eurasian Steppe was probably an extremely harsh place to live during the peak of the LGM [130]. While we do not argue that people abandoned this area completely, it is likely that this area was only scarcely occupied. Hence, after the peak of the LGM, the climate began to ameliorate but still had cold fluctuations. The microblade techno-complex could provide adaptive advantages to such environmental conditions and thus became prevalent across broad areas of the eastern Eurasian Steppe. Large areas of Siberia and Mongolia became inhabitable again due partly to the adoption of microblade technology which reduced subsistence risks [129]. This scenario reveals the complexities of the cultural and technological interactions between northern China and the eastern Eurasian Steppe, suggesting that northern China did not only accept introduced technology, but was also involved in the modification of transmitted technology and its subsequent transmission back into the eastern Eurasian Steppe. Notably, even though microblade technology emerged at the end of the Pleistocene in the Upper Paleolithic, it persistently proliferated across vast regions of the Mongolian Plateau, South Siberia, Northeast China and Northwest China into the Mid Holocene (including the Neolithic and the beginning of the Bronze Age). The long prevalence of microblade technology in such mid-high latitude continental parts of East Asia is likely due to its adaptative advantages for the intensified acquisition of animal resources [85].

### The nature and pathways of technological transmission

Whether the episodes of cultural transmission between the eastern Eurasian Steppe and northern China indicate direct population dispersal or cross-regional technological communication between different social groups remains unresolved. Equifinality exists because both of these behaviors would generate the cross-regional cultural similarities which we document in lithic assemblages. If we assume that population dispersal would lead to close similarities in lithic technologies between an original area and a new area, whereas technological communication between different groups would result in either intentional adjustments or unintentional changes to the original techno-complexes in the transmitted area, then the episodes of lithic transmission of the TMP, IUP, and LUP techno-complexes between the eastern Eurasian Steppe and northern China could be seen as resulting from population dispersal. In contrast, the development of microblade technology, based on the modification of MUP blade technology, could be characterized as the adoption of ideas. Local people adopted blade technology from the north and made their own modifications to increase suitability for local conditions. Admittedly, these are just tentative inferences rather than solid conclusions. Other lines of evidence, especially ancient DNA studies are needed to clarify whether similarities in technology might reflect dispersal of people, the uptake of ideas, or the combination of the two.

The site distributional pattern suggests that while the TMP/IUP techno-complex diffused into northern China from the west or northwest, the MUP blade technology probably diffused into North China from the north, along the corridors at the eastern edge of the Gobi-Steppe. The reason why technological transmission happened along different routes was probably also related to paleoenvironmental changes. While no obvious geographical barriers existed between the Eurasian Steppe and higher-latitude Northeast China, the desolate Gobi Desert situated between the core area of the Eurasian Steppe and North/Northwest China posed geographical obstacles for mutual cultural transmission. Only specific corridors comprising chains of oases could be passed through, which potentially served as important routes of technological communication. Therefore, the ameliorated MIS 3 period climatic conditions in arid Inner Asia facilitated southeastward cultural transmission from the west part of the eastern Eurasian Steppe into northern China.

Compared to the warmer and wetter MIS 3 period, the climatic change to colder and drier conditions during MIS 2 led to the expansion of dry desert areas situated between the eastern Eurasian Steppe and northern China [31, 149, 150]. This change would make the vast Gobi Desert less inhabitable and more difficult to pass through. Therefore, the relative wetter areas at the eastern margins of the Gobi Desert might become a more viable corridor for cultural transmission and the flow of people, ideas, and material culture across South Siberia, eastern Mongolia, and the eastern part of northern China.

## Conclusions

Episodes of cultural transmission can be identified between northern China and the eastern Eurasian Steppe after the Middle Paleolithic human migration into the latter region. Since the eastern Eurasian Steppe is culturally connected with western Eurasia in terms of lithic technology, the appearance of typical western lithic techno-complexes in northern China resulted from trans-regional technological transmission through the eastern Eurasian Steppe. From TMP and the beginning of the Upper Paleolithic, the Mousterian and blade technologies probably spread through the central Mongolian Steppe corridor into Northeast China, and through the foothills and along the oases corridor (across the Gobi Desert) into eastern Northwest China. The relatively wet and warm MIS 3 period ameliorated the environmental conditions in the dry Gobi Desert and facilitated cultural transmission from west to east. Nonetheless, both TMP Mousterian and IUP blade/Mousterian mixed techno-complexes were only confined to the northern periphery of northern China and did not penetrate into and replace the core-flake techno-complex in the hinterland of northern China. The higher costs of these sophisticated techno-complexes and the fact these technologies did not pose any real advantage over the simpler core-flake technology, given the environmental context, might explain why the transmitted Mousterian and blade technology did not thrive in northern China at this time.

Climatic changes to drier and colder conditions after MIS3 made the arid Gobi Desert increasingly impassable. In the MUP period, technological transmission probably occurred along the east margin of the Gobi Desert, connecting the eastern part of northern China and the eastern Eurasian Steppe. The spread of MUP blade technology between 28–25 ka BP impacted lithic technological development in a much broader region of northern China than the earlier TMP/IUP technology. Yet the broad spread of this technology was mainly achieved due to the development of microblade technology from the modification of previously introduced MUP blade technology, rather than the direct wholesale adoption of the MUP techno-complex in its original form. The microblade technology was well suited to the drier and colder environments of the LGM since it facilitated more efficient and intensive resource exploitation in high mobility contexts. As a result, it became further developed and gradually replaced the core-flake technology which once thrived across northern China.

The advantages of microblade technology for adapting to challenging environments might explain its widespread distribution across Northeast China and its eastern surroundings (like the Russian Far East, the Korean Peninsula, and Hokkaido) and vast regions of Mongolia and Siberia right after the peak of the LGM. This scenario suggests that people in northern China did not only receive lithic technologies from the eastern Eurasian steppe, but ultimately modified these technologies in ways which saw them subsequently spread back into the eastern Eurasian Steppe.

The various transmitted techno-complexes impacted different regions of northern China in different ways. In the north and west periphery of northern China, each episode of technological transmission can be detected, and the introduced techno-complex often, but not always remained in its original form, as it was close to the source area of diffusion. In contrast, less episodes of technological transmission left traces in the southern and central hinterland of northern China. In these regions, introduced technology was often modified from its original forms. Such a pattern suggests that distance from the eastern Eurasian Steppe and differences in ecological and geological conditions played important roles in shaping the distribution and adoption of waves of technological transmission between the eastern Eurasian Steppe and northern China.

Whether or not technological transmission between these two broad geographical units resulted from direct population dispersal or the exchange of ideas is open to discussion. We argue that both mechanisms, or some combination of them, could lead to the types of technological transmission evident in the archaeological record. Multidisciplinary research is needed to examine population migration, cultural interaction, technological evolution and how these processes related to patterns of cross-regional technological transmission between the eastern Eurasian Steppe and northern China.

## Supporting information

S1 Table The geographical coordinates, dating, and types of lithic technology of the sites discussed in the paper. The radiocarbon dates are calibrated by using OxCal 4.4 and the IntCal20 calibration curve. The references of the dating for each site are listed in S1 File.
(XLSX)

S1 File The bibliography of the sites discussed in the paper. The dating information and the identification of the types of lithic technology for each site discussed in the paper are based upon the references listed in this file.
(DOCX)
