# Supplementary material for: Diachronic shifts in lithic technological transmission between the eastern Eurasian Steppe and northern China in the Late Pleistocene
Source: PLoS One. 2022 Nov 3;17(11):e0275162. doi: 10.1371/journal.pone.0275162 (PMC9632798; doi:10.1371/journal.pone.0275162)
Supplement: S1 File — The dating information and the identification of the types of lithic technology for each site discussed in the paper are based upon the references listed in this file. (DOCX) [file pone.0275162.s002.docx]

**Bibliography of the sites discussed in the paper**

This file contains the references of the geographical location, dating results, and types of lithic technology of each site mapped in the article. The reference marked as “*” specifically indicates the one which provide dating information of each site listed on the “S1 Table: The geographical coordinates, dating, and types of lithic technology of the sites discussed in the paper”.

Below the name of each site, the figure and number of it mapped in the article is marked

**Okladnikov cave**

(Fig.3-1)

*Kuzmin YV, Orlova LA. Radiocarbon chronology of the Siberian Paleolithic. Journal of World Prehistory. 1998;12(1):1-53.

Derevianko AP, Powers WR, Shimkin DB. The Paleolithic of Siberia: New Discoveries and Interpretations (I.P. Larichva, Trans.). Novosibirsk: Institute of Archaeology and Ethnography, Siberian Division, Russian Academy of Sciences; 1998a.

**Strashnaya cave**

(Fig.3-2)

*Krivoshapkin A, Shalagina A, Baumann M, Shnaider S, Kolobova K. Between Denisovans and Neanderthals: Strashnaya Cave in the Altai Mountains. Antiquity. 2018;92(365):e1.

**Denisova cave**

(Fig.3-3)

*Morley MW, Goldberg P, Uliyanov VA, Kozlikin MB, Shunkov MV, Derevianko AP, et al. Hominin and animal activities in the microstratigraphic record from Denisova Cave (Altai Mountains, Russia). Scientific Reports. 2019;9(1):13785.

*Kuzmin YV, Orlova LA. Radiocarbon chronology of the Siberian Paleolithic. Journal of World Prehistory. 1998;12(1):1-53.

Shunkov MV, Kozlikin MB, Derevianko AP. Dynamics of the Altai Paleolithic industries in the archaeological record of Denisova Cave. Quaternary International. 2020;559:34-46.

**Ust Karakol**

(Fig.3-4)

* Derevianko AP, Agadzhanian AK, Baryshnikov GF, Dergacheva MI, Dupal TA, Malaeva EM, et al. Archaeology, Geology, and Paleogeography of the Pleistocene and Holocene of the Mountainous Altai (in Russian). Novosibirsk: Institute of Archaeology and Ethnography Press. 1998b.

*Derevianko AP, Postnov AV, Rybin EP, Kuzmin YV, Keates SG. The Pleistocene peopling of Siberia: a review of environmental and behavioral aspects. Bulletin of the Indo-Pacific Prehistory Association. 2005, 25:57e68.

**Kara-Tenesh**

(Fig.3-5)

*Kuzmin YV, Orlova LA. Radiocarbon Chronology of the Siberian Paleolithic. Journal of World Prehistory. 1998;12(1):1-53.

Zwyns N. Laminar technology and the onset of the Upper Paleolithic in the Altai Siberia. PhD dissertation, Leiden University; 2012.

**Tongtiandong**

(Fig.3-6)

* Yu J-J, Wang Y-P, He-J-N, Feng Y, Li Y-L, Li W-C. The Tongtiandong site in Jeminay County, Xinjiang (in Chinese). Kaogu. 2018(7):3-14.

**Mokhovo-2**

(Fig.3-7)

*Kuzmin YV, Orlova LA. Radiocarbon chronology of the Siberian Paleolithic. Journal of World Prehistory. 1998;12(1):1-53.

Rybin EP. Tools, beads, and migrations: Specific cultural traits in the Initial Upper Paleolithic of Southern Siberia and Central Asia. Quaternary International. 2014;347:39-52.

**Kharganyn Gol-5**

(Fig.3-8)

*Khatsenovich AM, Rybin EP, Gunchinsuren B, Olsen JW, Shelepaev RA, Zotkina LV, et al. New evidence for Paleolithic human behavior in Mongolia: The Kharganyn Gol 5 site. Quaternary International. 2017;442:78-94.

Rybin EP, Khatsenovich AM. Middle and Upper Paleolithic Levallois technology in eastern Central Asia. Quaternary International. 2020;535:117-138.

**Orkhon 1**

(Fig.3-9)

*Khatsenovich AM, Rybin EP, Bazargur D, Marchenko DV, Kogai SA, Shevchenko TA, et al. Middle Palaeolithic human dispersal in Central Asia: New archaeological investigations in the Orkhon Valley, Mongolia. Antiquity. 2019;93(370):e20.

**Jinsitai**

(Fig.3-10)

*Li F, Kuhn SL, Chen F-Y, Wang Y-H, Southon J, Peng F, et al. The easternmost Middle Paleolithic (Mousterian) from Jinsitai Cave, North China. Journal of Human Evolution. 2018;114:76-84.

**Sanlongdong cave**

(Fig.3-11)

Shan M-C, Narengova., Zhou X-Q, Chen F-Y. Discovery of 50,000-year-old Paleolithic Remains in Sanlongdong cave site, Inner Mongolia. Translated by Wang J [Internet]. Beijing: Chinese Arcaheology; 2018. Available from: http://www.kaogu.cn/en/News/New_discoveries/2018/0102/60610.html.

**Kara-Bom**

(Fig.3-12)

*Zwyns N, Rybin EP, Hublin JJ, Derevianko AP. Burin-core technology and laminar reduction sequences in the initial Upper Paleolithic from Kara-Bom (Gorny-Altai, Siberia). Quaternary International. 2012;259:33-47.

Belousova NE, Rybin EP, Fedorchenko AY, Аnoykin AA. Kara-Bom: new investigations of a Palaeolithic site in the Gorny Altai, Russia. Antiquity. 2018;92(361):e1.

Derevianko AP, Rybin EP. The earliest representations of symbolic behavior by Paleolithic humans in the Altai Mountains. Archaeology, Ethnology and Anthropology of Eurasia. 2003; 4 (3), 27e50.

**Luotuoshi**

(Fig.3-13)

*Derevianko AP, Xing G, Olsen JW, Rybin EP. The Paleolithic of Dzungaria (Xinjiang, Northwest China) Based on materials from the Luotuoshi Site. Archaeology, Ethnology and Anthropology of Eurasia. 2012;40(4):2-18.

**Chikhen Agui**

(Fig.3-14)

*Derevianko AP, Brantingham PJ, Olsen JW, Tseveendorj D. Initial Upper Paleolithic blade industries from the North-Central Gobi Desert, Mongolia. In: Brantingham PJ, Kuhn SL, Kerry KW, editors. The Early Upper Paleolithic beyond Western Europe. Berkely and Los Angeles: University of California Press; 2004. p. 207-222.

Brantingham PJ, Krivoshapkin Andrei I, Li J, Tserendagva Y. The Initial Upper Paleolithic in Northeast Asia. Current Anthropology. 2001;42(5):735-747.

**Tsagaan Agui**

(Fig.3-15)

*Derevianko AP, Brantingham PJ, Olsen JW, Tseveendorj D. Initial Upper Paleolithic blade industries from the North-Central Gobi Desert, Mongolia. In: Brantingham PJ, Kuhn SL, Kerry KW, editors. The Early Upper Paleolithic beyond Western Europe. Berkely and Los Angeles: University of California Press; 2004. p. 207-222.

**Voenny Hosptial**

(Fig.3-16)

*Goebel T. The Early Upper Paleolithic of Siberia. In: Brantingham PJ, Kuhn SL, Kerry KW, editors. The Early Upper Paleolithic beyond Western Europe. Berkeley and Los Angeles: University of California Press; 2004. p. 162-195.

**Kamenka**

(Fig.3-17)

*Kuzmin YV, Orlova LA. Radiocarbon Chronology of the Siberian Paleolithic. Journal of World Prehistory. 1998;12(1):1-53.

Zwyns N, Lbova LV. The Initial Upper Paleolithic of Kamenka site, Zabaikal region (Siberia): A closer look at the blade technology. Archaeological Research in Asia. 2019;17:24-49.

**Tolbaga**

(Fig.3-18 / Fig.8-15)

*Izuho M, Terry K, Vasil'ev S, Konstantinov M, Takahashi K. Tolbaga revisited: Scrutinizing occupation duration and its relationship with the faunal landscape during MIS 3 and MIS 2. Archaeological Research in Asia. 2019;17:9-23.

*Buvit I, Izuho M, Terry K, Konstantinov MV, Konstantinov AV. Radiocarbon dates, microblades and Late Pleistocene human migrations in the Transbaikal, Russia and the Paleo-Sakhalin-Hokkaido-Kuril Peninsula. Quaternary International. 2016; 425:100-119.

*Konstantinov MV. The Stone Age archaeological sites of the Baikal Asia (in Russian), Chita: Chita State Teacher Training College Press, 1994.

*Kuzmin YV, Orlova LA. Radiocarbon chronology of the Siberian Paleolithic. Journal of World Prehistory. 1998;12(1):1-53.

*Goebel T. The Middle to Upper Paleolithic Transition in Siberia. PhD dissertation, University of Alaska-Fairbanks,1993.

*Goebel T, Waters MR. New AMS 14C ages for the Tolbaga Upper Paleolithic site,Transbaikal, Siberia. Curr. Res. Pleistocene. 2000.17, 32–34.

Vasiliev SG, Rybin EP. Tolbaga: upper paleolithic settlement patterns in the trans- Baikal region. Archaeology, Ethnology and Anthropology of Eurasia. 2009;37(4):13-34.

**Tolbar-4**

(Fig.3-19)

*Rybin EP, Khatsenovich AM. Middle and Upper Paleolithic Levallois technology in eastern Central Asia. Quaternary International. 2020;535:117-138.

Gladyshev SA, Olsen JW, Tabarev AV, Kuzmin YV. Chronology and periodization of Upper Paleolithic sites in Mongolia. Archaeology, Ethnology and Anthropology of Eurasia. 2010;38(3):33-40.

Derevianko AP, Zenin AN, Rybin EP, Gladyshev SA, Tsybankov AA, Olsen JW, et al. The technology of early Upper Paleolithic lithic reduction in Northern Mongolia: The Tolbor-4 site. Archaeology, Ethnology and Anthropology of Eurasia. 2007;29(1):16-38.

**Nwya Devu**

(Fig.3-20)

*Zhang X-L, Ha B-B, Wang S-J, Chen Z-J, Ge J-Y, Long H, et al. The earliest human occupation of the high-altitude Tibetan Plateau 40 thousand to 30 thousand years ago. Science. 2018;362(6418):1049-1051.

**Lenghu**

(Fig.3-21)

*Brantingham PJ, Gao X. Peopling of the northern Tibetan Plateau. World Archaeology. 2006;38(3):387-414.

**Yushuwan**

(Fig.3-22)

*Zhang S-S. The Paleolithic remains found in the mid-south Inner Mongolia and north Shanxi (in Chinese). Vertebrata Palasiatica. 1958(1):34-43.

**Shuidonggou-1**

(Fig.3-23)

*Chen T-M, Yuan S-X, Gao S-J. The study on Uranium-series dating of fossil bones and an

absolute age sequence for the main Paleolithic sites of North China (in Chinese). Acta Anthropologica Sinica, 1984;3(3),259–269

*Morgan C, Barton L, Yi M-J, Bettinger RL, Gao X, Peng F. Redating Shuidonggou Locality 1 and implications for the Initial Upper Paleolithic in East Asia. Radiocarbon. 2014;56(1):165-179.

*Nian X-M, Gao X, Zhou L-P. Chronological studies of Shuidonggou (SDG) Locality 1 and their significance for archaeology. Quaternary International. 2014;347:5-11.

Peng F, Wang H-M, Gao X. Blade production of Shuidonggou Locality1 (Northwest China): A technological perspective. Quaternary International. 2014;347:12-20.

**Shuidonggou-2**

(Fig.3-24 / Fig.8-27)

*Liu D-C, Wang X-L, Gao X, Xia Z-K, Pei S-W, Chen F-Y, et al. Progress in the stratigraphy and geochronology of the Shuidonggou site, Ningxia, North China. Chinese Science Bulletin. 2009;54(21):3880.

*Li F, Kuhn SL, Gao X, Chen F-Y. Re-examination of the dates of large blade technology in China: A comparison of Shuidonggou Locality 1 and Locality 2. Journal of Human Evolution. 2013;64(2):161-168.

*Li F, Kuhn SL, Bar-Yosef O, Chen F-Y, Peng F, Gao X. History, Chronology and Techno-Typology of the Upper Paleolithic Sequence in the Shuidonggou Area, Northern China. Journal of World Prehistory. 2019;32(2):111-141.

*Madsen DB, Li J-Z, Brantingham PJ, Xing G, Elston RG, Bettinger RL. Dating Shuidonggou and the Upper Palaeolithic blade industry in North China. Antiquity. 2001;75(290):706-716.

**Changweigou**

(Fig.3-25)

*Xie J-Y. Retrospect and progress of Paleolithic archaeology in Gansu (in Chinese). In: Prehistory Research Institute, C.N.U., South Korea, and Archaeological Institute of Liaoning Province, editors, International Symposium on Paleolithic Culture in Northeastern Asia Including China, Russia and South Korea. 1997. p. 165–177.

**ZS08**

(Fig.3-26)

*Barton L, Brantingham PJ, Ji D-X. Late Pleistocene climate change and Paleolithic cultural evolution in northern China: Implications from the Last Glacial Maximum. Developments in Quaternary Sciences. 2007;9:105-128.

**Wulanmulun**

(Fig.3-27)

*Li X-Q, Gao Q, Hou Y-M, Zhao K-L, Sun N, Yang Z-M, et al. The vegetation and environment at the Wulamulun site in the Ordos Plateau, Inner Mongolia during MIS 3 period (in Chinese). Acta Anthropologica Sinica. 2014;33(1):60-69.

Lei L, Liu Y, Hou Y-M, Zhang J-F, Bao L, Hu Y, et al. A study of stone artifacts from 2014-2015 excavations at the Wulanmulun Locality 2, Ordos, Inner Mongolia (in Chinese). Acta Anthropologica Sinica. 2019;38(2):157-165.

**Longquandong Cave**

(Fig.3-28)

*Li X, Gu X-J, Pang H-J. The preliminary report of the 2014 excavation of the Paleolithic Longquan Cave site in Luanchuan, Henan (in Chinese). Kaogu yu Wenwu. 2017(6):3-12.

*Du S-S, Zhou L, Pang H-J, Li X, Wang J. The excavation of the Longquan Cave site in Luanchuan, Henan in 2011 (in Chinese). Kaogu Xuebao. 2017(2):227-252.

*Zhou L, Li X, Pang H-J, Du S-S. Preliminary analysis of lithic assemblage excavated in Longquan Cave, Henan Province, Central China (in Chinese). Quaternary Sciences. 2011;31(06):972-981.

**Zhijidong Cave**

(Fig.3-29)

*Wang Y-P. The lithic industry of the Zhiji Cave and early human activies (in Chinese). In: Peking University School of Archaeology and Museology, editor. Archaeological Research (Seventh). Beijing: China Science Publishing; 2008. p. 136-148.

Zhang S-L, Liu Y-F. Report on the excavation of Zhijidong Cave site (in Chinese). Acta Anthropologica Sinica. 2003;22(1):1-17.

**Fangjiagou**

(Fig.3-30)

*Peking University School of Archaeology and Musology, Zhengzhou Institute of Cultural Relics and Archaeology. Report on the Excavation of the Fangjiagou Site (in Chinese). Beijing: China Science Publishing; 2020.

**Zhaozhuang**

(Fig.3-31)

*Zhengzhou Institute of Cultural Relics and Archaeology, Peking University School of Archaeology and Musology. Report on the Excavation of the Xinzheng Zhaozhuang Site (in Chinese). Beijing: China Science Publishing; 2020.

**Laonainaimiao**

(Fig.3-32)

*Wang Y-P. The settlement pattern of the early humans in MIS 3 period of the southeastern foot of the Song Mountain (in Chinese). In: Peking University School of Archaeology and Museology, editor. Archaeological Research (Ten). Beijing: China Science Publishing; 2012. p. 287-296.

Chen Y-C, Qu T-L, Zhang S-L, Gu W-F, Wang S-Z, Wang Y-P. A preliminary research on core typology from the Laonainaimiao site in Zhengzhou, Central China (in Chinese). Acta Anthropologica Sinica. 2019;38(02):200-211.

**Xiachuan (Fuyuhe)**

(Fig.3-33)

*Chen, C., Wang, X.-Q. 1989. Upper Paleolithic microblade industries in North China and their relationships with northeast Asia and North America. Arctic Anthropology. 1989;26: 127–156.

**Xibaimaying**

(Fig.3-34)

*Guo Y-J, Li B, Zhang J-F, Yuan B-Y, Xie F, Roberts RG. New ages for the Upper Palaeolithic site of Xibaimaying in the Nihewan Basin, northern China: implications for small-tool and microblade industries in north-east Asia during Marine Isotope Stages 2 and 3. Journal of Quaternary Science. 2017;32(4):540-552.

*Zhou Z-Y, Wang F-G. The preliminary report on the 2015 trial excavation of the Xibaimaying Paleolithic site of the Yangyuan county, Hebei (in Chinese). Kaogu. 2019(10):3-14.

**Xigouwan**

(Fig.3-35)

*Xue F, Xiao Y-N, Leng Y-T, Li D-Y, Li Y-H, Niu D-W. The excavation of the Xigouwan Paleolithic Locality 1 in Huailai Basin (in Chinese). Acta Anthropologica Sinica. 2019;38(2):212-222.

**Xiaogushan**

(Fig.3-36)

*Huang W-W, Fu R-Y. The Comprehensive Research of the Xiaogushan Prehistoric Cave Site in Haicheng, Liaoning (in Chinese). Beijing: China Science Publishing; 2009.

**Mingyuegou**

(Fig.3-37)

*IA-CASS (The Institute of Archaeology – Chinese Academy of Social Sciences). Radiocarbon Dates in Chinese Archaeology, 1965–1991 (in Chinese). Beijing: Cultural Relics Publishing House, 1991.

Barton L, Brantingham PJ, Ji D-X. Late Pleistocene climate change and Paleolithic cultural evolution in northern China: Implications from the Last Glacial Maximum. Developments in Quaternary Sciences. 2007; 9: 105-128.

**Zhoujiayoufang**

(Fig.3-38)

*IA-CASS (The Institute of Archaeology – Chinese Academy of Social Sciences). Radiocarbon Dates in Chinese Archaeology, 1965–1991 (in Chinese). Beijing: Cultural Relics Publishing House, 1991.

Sun J-Z, Wang Y-Z, Jiang P. A Paleolihtic site at Zhou-Jia-You-Fang in Yushu county, Jilin province (in Chinese). Vertebrata Palasiatica. 1981;19(3):281-291.

**Guxiangtun**

(Fig.3-39)

*IA-CASS (The Institute of Archaeology – Chinese Academy of Social Sciences). Radiocarbon Dates in Chinese Archaeology, 1965–1991 (in Chinese). Beijing: Cultural Relics Publishing House, 1991.

Wang Y-P. The Origin of Ancient Human Culture in China (in Chinese). Beijing: China Science Publishing; 2005.

**Zhailainuoer**

(Fig.3-40)

*IA-CASS (The Institute of Archaeology – Chinese Academy of Social Sciences). Radiocarbon Dates in Chinese Archaeology, 1965–1991 (in Chinese). Beijing: Cultural Relics Publishing House, 1991.

Wang Y-H, Sun Z-D, Shan M-C, He J, Liu W, Chen F-Y. A preliminary report on the survey and test excavation of the Mogushanbei site in Zhalainuoer district, Inner Mongolia（in Chinese). Acta Anthropologica Sinica. 2020;39(2):173-182.

**Shiyu**

(Fig.3-41)

*IA-CASS (The Institute of Archaeology – Chinese Academy of Social Sciences). Radiocarbon Dates in Chinese Archaeology, 1965–1991 (in Chinese). Beijing: Cultural Relics Publishing House, 1991.

*Yuan S-X. AMS radiocarbon dating of Xinglong carved antler, Shiyu and Ximiao sites (in Chinese). Acta Anthropologica Sinica. 1993;(1):92-95.

Jia L-P, Gai P, You Y-Z. The excavation report on the Shiyu Paleolithic site of Shanxi Province (in Chinese). Kaogu Xuebao. 1972;(1):39-58.

**Zhoukoudian Upper Cave**

(Fig.3-42)

*Chen T-M, Hedges REM, Yuan Z-X. The second batch of accelerator radiocarbon dates for Upper Cave site of Zhoukoudian (in Chinese). Acta Anthropologica Sinica. 1992;11(2):112-116.

Qu T-L, Bar-Yosef O, Wang Y-P, Wu X-H. The Chinese Upper Paleolithic: Geography, Chronology, and Techno-typology. Journal of Archaeological Research. 2013;21(1):1-73.

**Anui-2**

(Fig.8-1)

*Kuzmin YV, Orlova LA. Radiocarbon Chronology of the Siberian Paleolithic. Journal of World Prehistory. 1998;12(1):1-53.

Derevianko AP, Powers WR, Shimkin DB. The Paleolithic of Siberia: New Discoveries and Interpretations (I.P. Larichva, Trans.). Novosibirsk: Institute of Archaeology and Ethnography, Siberian Division, Russian Academy of Sciences; 1998a.

**Ui-1**

(Fig.8-2)

*Graf KE. “The Good, the Bad, and the Ugly”: evaluating the radiocarbon chronology of the middle and late Upper Paleolithic in the Enisei River valley, south-central Siberia. Journal of Archaeological Science. 2009;36(3):694-707.

Vasil'ev SA. The Late Paleolithic of the Yenisei: A new outline. Journal of World Prehistory. 1992;6(3):337-383.

**Sabanikha**

(Fig.8-3)

*Graf KE. “The Good, the Bad, and the Ugly”: evaluating the radiocarbon chronology of the middle and late Upper Paleolithic in the Enisei River valley, south-central Siberia. Journal of Archaeological Science. 2009;36(3):694-707.

Graf KE. Hunter–gatherer dispersals in the mammoth-steppe: technological provisioning and land-use in the Enisei River valley, south-central Siberia. Journal of Archaeological Science. 2010;37(1):210-223.

**Novoselovo-13**

(Fig.8-4)

*Vasil'ev SA, Kuzmin YV, Orlova LA, Dementiev VN. Radiocarbon-based chronology of the Paleolithic in Siberia and its relevance to the peopling of the New World. Radiocarbon. 2002;44(2):503-530.

Vasil'ev SA. The Late Paleolithic of the Yenisei: A new outline. Journal of World Prehistory. 1992;6(3):337-383.

**Kurtak-4**

(Fig.8-5)

*Vasil'ev SA, Kuzmin YV, Orlova LA, Dementiev VN. Radiocarbon-based chronology of the Paleolithic in Siberia and its relevance to the peopling of the New World. Radiocarbon. 2002;44(2):503-530.

Graf KE. Hunter–gatherer dispersals in the mammoth-steppe: technological provisioning and land-use in the Enisei River valley, south-central Siberia. Journal of Archaeological Science. 2010;37(1):210-223.

**Derbina-5**

(Fig.8-6)

*Kuzmin YV, Keates SG. Siberia and neighboring regions in the Last Glacial Maximum: did people occupy northern Eurasia at that time? Archaeological and Anthropological Sciences. 2018;10(1):111-124.

Graf KE, Buvit I. Human dispersal from Siberia to Beringia: Assessing a Beringian standstill in light of the archaeological evidence. Current Anthropology. 2017;58(S17):S583-S603.

**Malta**

(Fig.8-7)

*Medvedev G, Cauwe N, Vorob’eva G, Coupe D, Claes L, Lipnina E, et al. The Malta Paleolithic Locality (in Russian). 2016; Irkutsk: ARCOM Press.

Derevianko AP, Powers WR, Shimkin DB. The Paleolithic of Siberia: New Discoveries and Interpretations (I.P. Larichva, Trans.). Novosibirsk: Institute of Archaeology and Ethnography, Siberian Division, Russian Academy of Sciences; 1998a.

**Buret'**

(Fig.8-8)

*Vasil'ev SA, Kuzmin YV, Orlova LA, Dementiev VN. Radiocarbon-based chronology of the Paleolithic in Siberia and its Relevance to the peopling of the New World. Radiocarbon. 2002;44(2):503-30.

Derevianko AP, Powers WR, Shimkin DB. The Paleolithic of Siberia: New Discoveries and Interpretations (I.P. Larichva, Trans.). Novosibirsk: Institute of Archaeology and Ethnography, Siberian Division, Russian Academy of Sciences; 1998a.

**Chitkan**

(Fig.8-9)

*Buvit I, Izuho M, Terry K, Konstantinov MV, Konstantinov AV. Radiocarbon dates, microblades and Late Pleistocene human migrations in the Transbaikal, Russia and the Paleo-Sakhalin-Hokkaido-Kuril Peninsula. Quaternary International. 2016; 425:100-119.

Terry K, Buvit I, Konstantinov MV. Emergence of a microlithic complex in the Transbaikal Region of southern Siberia. Quaternary International. 2016; 425:88-99.

**Krasny Yar-1**

(Fig.8-10)

*Kuzmin YV, Keates SG. Siberia and neighboring regions in the Last Glacial Maximum: did people occupy northern Eurasia at that time? Archaeological and Anthropological Sciences. 2018;10(1):111-124.

Derevianko AP, Powers WR, Shimkin DB. The Paleolithic of Siberia: New Discoveries and Interpretations (I.P. Larichva, Trans.). Novosibirsk: Institute of Archaeology and Ethnography, Siberian Division, Russian Academy of Sciences; 1998a.

**Shishkino-8**

(Fig.8-11)

*Kuzmin YV, Keates SG. Siberia and neighboring regions in the Last Glacial Maximum: did people occupy northern Eurasia at that time? Archaeological and Anthropological Sciences. 2018;10(1):111-124.

Aksenov MP. Pre-Neolithic sites at the Kachug-Upper Lena portion of the Upper Lena valley (in Russian). In: Medvedev GI, editor. Arkheologicheskoye nasledie Baikalskoi Sibiri. Part 1. Irkutsk: Center for Cultural Heritage Preservation; 1996. p.12–22.

**Dorolj-1**

(Fig.8-12)

*Jaubert J, Bertran P, Fontugne M, Jarry M, Lacombe S, Leroyer C, et al. Le Paleolithique superieur ancien de Mongolie: Dorolj 1(Egiïn Gol). Analogies avec les donnees de l'Altaï et de Siberie. In: Le Secretariatdu Congres, editor, The Upper Palaeolithic General Sessions and Posters. Acts of the XIVth UISPP Congress, University of Liege, Belgium, 2004; 2-8 September 2001.Archaeopress, Oxford. 2004. p. 245e251.

Rybin EP, Khatsenovich AM, Gunchinsuren B, Olsen JW, Zwyns N. The impact of the LGM on the development of the Upper Paleolithic in Mongolia. Quaternary International. 2016;425:69-87.

**Orkhon-7**

(Fig.8-13)

*Astashkin AV, Derevianko AP, Milov AD, Nikolaev SV, Petrin VT, Tzvetkov YD. The EPR-dating: comparison of EPR and 14C methods in bone dating at the archaeological site Orkhon-7 (Mongolia). Altaica 3, 1993; 9-15.

Rybin EP, Khatsenovich AM, Gunchinsuren B, Olsen JW, Zwyns N. The impact of the LGM on the development of the Upper Paleolithic in Mongolia. Quaternary International. 2016;425:69-87.

**Priiskovoe**

(Fig.8-14)

*Buvit I, Izuho M, Terry K, Konstantinov MV, Konstantinov AV. Radiocarbon dates, microblades and Late Pleistocene human migrations in the Transbaikal, Russia and the Paleo-Sakhalin-Hokkaido-Kuril Peninsula. Quaternary International. 2016; 425:100-19.

*Goebel T. The Middle to Upper Paleolithic Transition in Siberia. PhD dissertation, University of Alaska-Fairbanks,1993.

Terry K, Buvit I, Konstantinov MV. Emergence of a microlithic complex in the Transbaikal Region of southern Siberia. Quaternary International. 2016; 425:88-99.

**Alekseevsk**

(Fig.8-16)

*Kuzmin YV, Keates SG. Siberia and neighboring regions in the Last Glacial Maximum: did people occupy northern Eurasia at that time? Archaeological and Anthropological Sciences. 2018;10(1):111-124.

Goebel T. Pleistocene human colonization of Siberia and peopling of the Americas: An ecological approach. Evolutionary Anthropology. 1999;8(6):208-227.

**Xishantou**

(Fig.8-17)

*Liu W, Li Y-Q, Yang S-X. The trial excavation of the Xishantou site of the Paleolithic age in Longjiang County, Heilongjiang Province (in Chinese). Kaogu. 2019(11):3-13.

Yue J-P, Yang S-X, Li Y-Q, Storozum M, Hou Y-M, Chang Y, et al. Human adaptations during MIS 2: Evidence from microblade industries of Northeast China. Palaeogeography, Palaeoclimatology, Palaeoecology. 2021; 567:110286.

**Youfang**

(Fig.8-18)

*Nian X-M, Gao X, Xie F, Mei H-J, Zhou L-P. Chronology of the Youfang site and its implications for the emergence of microblade technology in North China. Quaternary International. 2014; 347:113-121.

Xie F, Cheng S-Q. Report on the excavation of microliths site at Youfang, Yangyuan county, Hebei Province (in Chinese). Acta Anthropologica Sinica. 1989;08(01):59-68.

**Xishahe**

(Fig.8-19)

*Guan Y, Wang X, Wang F, Olsen JW, Pei S, Zhou Z, et al. Microblade remains from the Xishahe site, North China and their implications for the origin of microblade technology in Northeast Asia. Quaternary International. 2020; 535:38-47.

**Shizitan-29**

(Fig.8-20)

*Song Y-H, Cohen DJ, Shi J-M, Wu X-H, Kvavadze E, Goldberg P, et al. Environmental reconstruction and dating of Shizitan 29, Shanxi Province: An early microblade site in north China. Journal of Archaeological Science. 2017;79:19-35.

Song Y, Shi J. The excavation of the Locality S29 of Shizitan Paleolithic site in Jixian County, Shanxi (in Chinese). Kaogu. 2017(02):35-51.

Song Y-H, Grimaldi S, Santaniello F, Cohen DJ, Shi J-M, Bar-Yosef O. Re-thinking the evolution of microblade technology in East Asia: Techno-functional understanding of the lithic assemblage from Shizitan 29 (Shanxi, China). PLOS ONE. 2019;14(2):e0212643.

**Longwangchan**

(Fig.8-21)

*Wang X-Q, Zhang J-F. Processing technology and the chronology of the microlith in the Longwangchan Loc. 1 - An extended discussion on the origin of Mirolith in North China (in Chinese). Nanfang Wenwu. 2016(4):49-56.

*Zhang J-F, Wang X-Q, Qiu W-L, Shelach G, Hu G, Fu X, et al. The paleolithic site of Longwangchan in the middle Yellow River, China: chronology, paleoenvironment and implications. Journal of Archaeological Science. 2011;38(7):1537-1550.

**Xiachuan (Xiaobaihuageliang, Locality 1, Locality 2)**

(Fig.8-22)

*Du S-S, Wang J, Wang Y-R, Shan Y-Y. The excavation of the Xiaobaihuageliang locality of Xiachuan site in Qingshui Shanxi in 2015 (in Chinese). Acta Anthropologica Sinica. 2019(3):383-408.

Du S-S. Continuity and break: Rethinking the significance of Xiachuan site in Chinese Paleolithic research (in Chinese). Quaternary Sciences. 2021;41(1):153-163.

*Chen C, Wang X-Q. Upper Paleolithic microblade industries in North China and their relationships with Northeast Asia and North America. Arctic Anthropology. 1989;26(2):127-156.

**Xishi / Dongshi**

(Fig.8-23)

*Zhao C, Wang Y-P, Gu W-F, Wang S-Z, Wu X-H, Gao X-X, et al. The emergence of early microblade technology in the hinterland of North China: a case study based on the Xishi and Dongshi site in Henan Province. Archaeological and Anthropological Sciences. 2021;13(6):98.

Wang Y-P, Wang S-Z, Zhao C, Chen Y-C. The excavation of the Upper Paleolithic Dongshi site at Dengfeng, Henan in 2013 (in Cinese). Zhongyuan Wenwu. 2018(6):46-53.

**ZL-05**

(Fig.8-24)

*Barton L, Brantingham PJ, Ji D-X. Late Pleistocene climate change and Paleolithic cultural evolution in northern China: Implications from the Last Glacial Maximum. Developments in Quaternary Sciences. 2007;9:105-128.

Ji D-X, Chen F-H, Bettinger RI, Elston R, Geng Z-Q, Barton LM, et al. Human Response to the Last Glacial Maximum: Evidence from North China (in Chinese). Acta Anthropologica Sinica. 2005(4):270-282.

*Zhang D, Chen F, Ji D, Barton L, Brantingham PJ, Wang H. The age, lithics and paleoenvironmental study of the Sumiaoyuantou Locality, Gansu Province (in Chinese). Acta Anthropologica Sinica. 2011;30(3):289-298.

**TX08**

(Fig.8-25)

*Ji D-X, Chen F-H, Bettinger RI, Elston R, Geng Z-Q, Barton L, et al. Human response to the Last Glacial Maximum: Evidence from North China (in Chinese). Acta Anthropologica Sinica. 2005(04):270-282.

**TX03**

(Fig.8-26)

*Ji D-X, Chen F-H, Bettinger RI, Elston R, Geng Z-Q, Barton L, et al. Human response to the Last Glacial Maximum: Evidence from North China (in Chinese). Acta Anthropologica Sinica. 2005(04):270-282.

**Tashuihe**

(Fig.8-28)

*IA-CASS (The Institute of Archaeology – Chinese Academy of Social Sciences). 1992. Radiocarbon dating report 19. Kaogu. 1992(7): 655–672.

Chen Z-Y. The Paleolithic remains of the Tashuihe, Lingchuan (in Chinese). Wenwu Jikan. 1989(02):97-98.

Du S-S. A study of the lithic assemblages of the Tashuihe site in Lingchuan, Shanxi (in Chinese). Kaogu yu Wenwu. 2007(4):86-93.

**Xiaonanhai**

(Fig.8-29)

*Lu T-L-D. The Transition from Foraging to Farming and the Origin of Agriculture in China. Oxford: BAR International Series 774. British Archaeological Reports; 1999.

An Z-M. The test excavation of the Paleolithic cave deposition of the Xiaonanhai site in Anyang, Henan (in Chinese). Kaogu Xuebao. 1965(1):1-27.

**Dongfangguangchang**

(Fig.8-30)

*Li C-R, Yu J-C, Feng X-W. A brief report on the excavation of the Paleolithic site of the Dongfangguangchang, Wangfujin in Beijing City (in Chinese). Kaogu. 2000(9):1-18.

Feng X-W, Li C-R, Yu J-C. A study on the stone artifacts from the Orient Plaza site of Beijing (in Chinese). Acta Anthropologica Sinica. 2006;25(4):285-298.

**Miaohoushan**

(Fig.8-31)

*IA-CASS (The Institute of Archaeology – Chinese Academy of Social Sciences). Radiocarbon dates in Chinese Archaeology, 1965–1991 (in Chinese). Beijing: Cultural Relics Publishing House, 1991.

Liaoning Provincial Museum, Benxi Museum. Miaohoushan: The Paleolithic Site of Benxi City, Liaoning Province (in Chinese). Beijing: Cultural Relics Press, 1986.

**Xuetian**

(Fig.8-32)

*Fu R. Paleolithic cultures and their characteristics in northeast China (in Chinese). Paper presented at the International Symposium Commemorating the 80^th^ Anniversary of the Discovery of Shuidonggou. Yinchuan, Ningxia Province, P.R.C. 2003.

Yu H-L. A brief study of late Paleolithic localities at Xuetian village of Wuchang County, Heilongjiang Province (in Chinese). Acta Anthropologica Sinica. 1988;7(3):255-263.

**Yanjiagang**

(Fig.8-33)

*Wu X-Z, Poirier FE. Human Evolution in China: A Metric Description of the Fossils and a Review of the Sites. New York: Oxford University Press. 1995.

Wei Z-Y, Yang D-S, Yin K-P, Nie Q-X, Yu H-L. The Upper Paleolithic locality of Yanjiagang in Harbin (Excavation report of the year 1982-1983) (in Chinese). Beifang Wenwu. 1986(4):8-15.

**Shibazhan**

(Fig.8-34)

*Zhang X-L, Yu H-L, Gao X. New findings and dates of the Shibazhan Paleolithic site, Heilongjiang Province (in Chinese). Acta Anthropologica Sinica. 2006;25(2):115-128.

Wei Z-Y, Gan Z-G. The new findings of the Paleolithic remains in Huma Shibazhan (in Chinese). Qiushi Xuekan. 1981(1):118-121.

**Ust- Ulma**

(Fig.8-35)

*Kuzmin YV. Siberia at the Last Glacial Maximum: Environment and Archaeology. Journal of Archaeological Research. 2008;16(2):163-221.

Keates SG, Postnov AV, Kuzmin YV. Towards the origin of microblade technology in northeastern Asia. Vestnik of Saint Petersburgh University, History. 2019;64(2):390-414.

**Dadong**

(Fig.8-36 / Fig.9-21)

*Li W-B, Chen Q-J, Fang Q, Zhao H-L. A preliminary report on the trial excavation at Dadong Paleolithic site in Helong, Yanbian, Jilin Province, 2007 (in Chinese). Research of China's Frontier Archaeology. 2016(2):1-11.

Yue J-P, Yang S-X, Li Y-Q, Storozum M, Hou Y-M, Chang Y, et al. Human adaptations during MIS 2: Evidence from microblade industries of Northeast China. Palaeogeography, Palaeoclimatology, Palaeoecology. 2021;567:110286.

**Janheung-ri**

(Fig.8-37 / Fig.9-22)

*Kim JC, Youn MY, Kim IC, Park JH, Song YM, Kang J, et al. Dating paleosols from Paleolithic sites in Korea. Nuclear Instruments and Methods in Physics Research Section B: Beam Interactions with Materials and Atoms. 2004;223-224:723-30.

Gómez Coutouly YA. The emergence of pressure knapping microblade technology in Northeast Asia. Radiocarbon. 2018;60(3):821-55.

**Hopyeong-dong**

(Fig.8-38 / Fig.9-23)

*Hong M-Y, Kim J-H. Hopyeongdong Paleolithic site (Namyangju, Gyeonggi Province, Korea) II. Excavation Report N°93 (in Korean). Seoul: Korea Land Corporation, Gyeonggi Cultural Foundation and Gijeon Institute of Cultural Properties. 2008.

Bae KD. Origin and patterns of the Upper Paleolithic industries in the Korean Peninsula and movement of modern humans in East Asia. Quaternary International. 2010;211(1-2):103-12.

**Daejeong-dong**

(Fig.8-39 / Fig.9-25)

*Seong C. Tanged points, microblades and Late Palaeolithic hunting in Korea. Antiquity. 2008;82(318):871-883.

Seong C. Late Pleistocene microlithic assemblages in Korea. In: Kuzmin YV, Keates Susan G, Chen S, editors. Origin and Spread of Microblade Technology in Northern Asia and North America. Burnaby (BC): Archaeology Press, Simon Fraser University. 2007. p 103–144.

**Sinbuk**

(Fig.8-40 / Fig.9-26)

*Kim JC, Kim DK, Youn M, Yun CC, Park G, Woo HJ, Hong M-Y, Lee GK. Pixe Provenance of Obsidian Artefacts from Paleolithic Sites in Korea. Indo-Pacific Prehistory Association Bulletin 2007; 27:122–128.

Gómez Coutouly YA. The emergence of pressure knapping microblade technology in Northeast Asia. Radiocarbon. 2018;60(3):821-855.

**Pirika-1**

(Fig.8-42 / Fig.9-18)

*Naganuma T. Pirika 1 Site (in Japanese). Sapporo: Hokkaido Archaeological Research Center. 1985.

**Kashiwaidai-1**

(Fig.8-41 / Fig.9-19)

*Nakazawa Y, Izuho M, Takakura J, Yamada S. Toward an Understanding of Technological Variability in Microblade Assemblages in Hokkaido, Japan. Asian Perspectives. 2005;44(2):276-292.

Iwase A. A functional analysis of the LGM microblade assemblage in Hokkaido, northern Japan: A case study of Kashiwadai 1. Quaternary International. 2016;425:140-157.

**Ogonki-5**

(Fig.8-43 / Fig.9-17)

*Kuzmin YV. Siberia at the Last Glacial Maximum: Environment and Archaeology. Journal of Archaeological Research. 2008;16(2):163-221.

Vasilevski AA. Periodization and classification of the Upper Paleolithic of Sakhalin and Hokkaido in the light of the research conducted at the Ogonki-5 site. Archaeology, Ethnology and Anthropology of Eurasia. 2003; 4(3):51–69.

**Mengjiaquan**

(Fig.9-1)

*Lu T-L-D. The Transition from Foraging to Farming and the Origin of Agriculture in China. Oxford: BAR International Series 774. British Archaeological Reports; 1999.

Xie F, Meng Z-Y, Wang Z-Y. A preliminary report on the excavation of the Mengjiaquan Paleolithic site in Hebei Province (in Chinese). Wenwu Chunqiu. 1991(01):1-13.

**Erdaoliang**

(Fig.9-2)

*Li G, Ren X-Y, Li J. A preliminary report on the excavation of Erdaoliang Upper Paleolithic site in the Nihewan Basin (in Chinese). Acta Anthropologica Sinica. 2014;34(2):509-521.

**Lingjing**

(Fig.9-3)

*Li Z-Y, Xing Y, Wu X-L, Li Y-N, Kato S, Zhao Q-P. The 2008-2013 excavation report on the level 5 of the Xuchang Man site, Lingjing (in Chinese). Huaxia Kaogu. 2018(2):3-33.

Li Z-Y, Li Y-N, Kato S. Observations of microblade core technologies from Level 5 of the Xuchang Man site, Lingjing (in Chinese). Acta Anthropologica Sinica. 2014;33(3):285-303.

**Xiachuan (Shunwangping, Shanshanyan)**

(Fig.9-4)

*Chen C, Wang X-Q. Upper Paleolithic microblade industries in North China and their relationships with Northeast Asia and North America. Arctic Anthropology. 1989;26(2):127-156.

*Lu T-L-D. The Transition from Foraging to Farming and the Origin of Agriculture in China. Oxford: BAR International Series 774. British Archaeological Reports; 1999.

IA-CASS (The Institute of Archaeology – Chinese Academy of Social Sciences), Shanxi Provincial Institute of Archaeology. The Excavation Report on the Upper Paleolithic Site of Xiachuan (in Chinese). Beijing: China Science Publishing. 2016.

**Xueguan**

(Fig.9-5)

*Lu T-L-D. The Transition from Foraging to Farming and the Origin of Agriculture in China. Oxford: BAR International Series 774. British Archaeological Reports; 1999.

Wang X-Q, Ding J-P, Tao F-H. Microliths from Xueguan, Puxian County, Shanxi (in Chinese). Acta Anthropologica Sinica. 1983(2):162-171.

**Shizitan-1**

(Fig.9-6)

*Yuan S-X, Zhao C-H, Zhu X-D, Yan J-Z, Yan Y-M. The chronological and cultural study of the Shizitan site, Jixian County, Shanxi (in Chinese). Kaogu. 1998(6):57-62.

Xie X-G, Yan J-Z, Tao F-H. The Mesolithic site at Shizitan, Jixian County, Shanxi Province (in Chinese). Kaogu Xuebao. 1989(3):305-323.

**Mengjiazhuang**

(Fig.9-7)

* Liu J-Z, Wang T-M, Jia W-L, Chen Z-Y. Microlithic remains from Yushe County, Shanxi Province (in Chinese). Acta Anthropologica Sinica. 1995;14(3):206-218.

**Shixiakou**

(Fig.9-8)

*Li F, Chen F-Y, Gao X, Liu D-C, Zhang D-J, Wang S. A Report on the 2009 Reconnaissance of Paleolithic remains in the Shuiluo and Qingshui River Valley, Gansu Province (in Chinese). Acta Anthropologica Sinica. 2011;30(2):137-148.

*Morgan C, Barton L, Bettinger R, Chen F, Dongju Z. Glacial cycles and Palaeolithic adaptive variability on China's Western Loess Plateau. Antiquity. 2011;85(328):365-379.

Ren J-C, Zhou J, Li F, Chen F-Y, Gao X. A preliminary report on 2015’s excavation at Shixiakou Paleolithic Locality 1 in Gansu Province, North China (in Chinese). Acta Anthropologica Sinica. 2017;36(1):1-17.

**PY-03**

(Fig.9-9)

*Ji D-X, Chen F-H, Bettinger RI, Elston R, Geng Z-Q, Barton LM, et al. Human response to the Last Glacial Maximum: Evidence from North China (in Chinese). Acta Anthropologica Sinica. 2005(4):270-282.

**Pigeon Mtn**

(Fig.9-10)

*Elston RG, Cheng X, Madsen DB, Kan Z, Bettinger RL, Jingzen L, et al. New dates for the north China Mesolithic. Antiquity. 1997;71(274):985-93.

*Madsen DB, Jingzen L, Elston RG, Cheng X, Bettinger RL, Kan G, et al. The Loess/Paleosol record and the nature of the younger Dryas climate in central China. Geoarchaeology.1998;13(8):847-69.

**Shuidonggou-12**

(Fig.9-11)

*Liu D-C, Chen F-Y, Zhang X-L, Pei S-W, Gao X, Xia Z-K. Preliminary comments on the paleoenvironment of the Shuidonggou Locality 12 (in Chinese). Acta Anthropologica Sinica. 2008(4):295-303.

Yi M, Barton L, Morgan C, Liu D, Chen F, Zhang Y, et al. Microblade technology and the rise of serial specialists in north-central China. Journal of Anthropological Archaeology. 2013;32(2):212-23.

**Hutouliang**

(Fig.9-12)

*Zhu Z-Y. The Study on Lithic Assemblages from the Hutouliang Site in North China (in Chinese). PhD dissertation. Institute of Vertebrate Paleontology and Paleoanthropology. 2006.

Zhu Z-Y, Gao X. Microlithic technology from Hutouliang site (in Chinese). Acta Anthropologica Sinica. 2007;26(04):305-310.

Gai P, Wei Q. Discovery of the Late Paleolithic site at Hutouliang, Hebei (in Chinese). Vertebtata Palasiatica. 1977(4):287-300.

**Maanshan**

(Fig.9-13)

*Gao L. A Study on lithic artifacts unearthed from Layer 3 and 4 of Maanshan 2, Yangyuan County, Hebei Province (in Chinese). MA thesis. Northwest University of China; 2016.

**Taoshan**

(Fig.9-14)

*Yue J-P, Hou Y-M, Yang S-X, Chang Y, Zhhang W, Li Y-Q, et al. A preliminary report on the 2014 excavation at Taoshan site in Heilongjiang Province, Northeast China. Acta Anthropologica Sinica. 2017;36(2):180-192.

Yue J-P, Li Y-Q, Yang S-X. Neolithisation in the southern Lesser Khingan Mountains: lithic technologies and ecological adaptation. Antiquity. 2019;93(371):1144-1160.

**Huayang**

(Fig.9-15)

*Yue J-P, Li Y-Q, Yang S-X. Neolithisation in the southern Lesser Khingan Mountains: lithic technologies and ecological adaptation. Antiquity. 2019;93(371):1144-1160.

**Suvorovo-4**

(Fig.9-20)

*Vasil'ev SA, Kuzmin YV, Orlova LA, Dementiev VN. Radiocarbon-based chronology of the Paleolithic in Siberia and its relevance to the peopling of the New World. Radiocarbon. 2002;44(2):503-30.

Kuzmin YV. People and environment in the Russian far east from Paleolithic to Middle Ages: chronology, palaeogeography, interaction. GeoJournal. 1995;35(1):79-83.

**Suyanggae**

(Fig.9-24)

*Yi Y-J. Excavation Report on Danyang Suyanggae Paleolithic Site (in Korean). Cheongju: Chungbuk National University Museum, 1984.

*Lee Y-J, Kong S. Le site Paléolithique de Suyanggae, Corée. L’anthropologie. 2006;110: 223–240.

**Ust-Menza-2**

(Fig.9-27)

*Konstantinov MV. The Stone Age archaeological sites of the Baikal Asia (in Russian). Chita: Chita State Teacher Training College Press, 1994.

Goebel T. The “Microblade Adaptation” and recolonization of Siberia during the Late Upper Pleistocene. In: Elston RG, Kuhn SL, editors. Thinking Small: Global Perspectives on Microlithization. Arlington: American Anthropological Association; 2002. p. 117-132.

**Studenoe-2**

(Fig.9-28)

*Goebel T, Waters MR, Buvit I, Konstantinov MV, Konstantinov AV. Studenoe-2 and the origins of microblade technologies in the Transbaikal, Siberia. Antiquity. 2000;74(285):567-575.

Buvit I, Izuho M, Terry K, Konstantinov MV, Konstantinov AV. Radiocarbon dates, microblades and Late Pleistocene human migrations in the Transbaikal, Russia and the Paleo-Sakhalin-Hokkaido-Kuril Peninsula. Quaternary International. 2016; 425:100-119.

**Sokhatino-4**

(Fig.9-29)

*Vasil'ev SA, Kuzmin YV, Orlova LA, Dementiev VN. Radiocarbon-based chronology of the Paleolithic in Siberia and its relevance to the peopling of the New World. Radiocarbon. 2002;44(2):503-30.

Goebel T. The “Microblade Adaptation” and recolonization of Siberia during the Late Upper Pleistocene. In: Elston RG, Kuhn SL, editors. Thinking Small: Global Perspectives on Microlithization. Arlington: American Anthropological Association; 2002. p. 117-32.

Derevianko AP, Powers WR, Shimkin DB. The Paleolithic of Siberia: New Discoveries and Interpretations (I.P. Larichva, Trans.). Novosibirsk: Institute of Archaeology and Ethnography, Siberian Division, Russian Academy of Sciences; 1998a.

**Tolbar-15**

(Fig.9-30)

*Gladyshev SA, Olsen JW, Tabarev AV, Kuzmin YV. Chronology and periodization of Upper Paleolithic sites in Mongolia. Archaeology, Ethnology and Anthropology of Eurasia. 2010;38(3):33-40.

Gladyshev SA, Olsen JW, Tabarev AV, Jull AJT. The Upper Paleolithic of Mongolia: Recent finds and new perspectives. Quaternary International. 2012; 281:36-46.

**Kurla-3**

(Fig.9-31)

*Vasil'ev SA, Kuzmin YV, Orlova LA, Dementiev VN. Radiocarbon-based chronology of the Paleolithic in Siberia and its relevance to the peopling of the New World. Radiocarbon. 2002;44(2):503-530.

Goebel T. The “Microblade Adaptation” and recolonization of Siberia during the Late Upper Pleistocene. In: Elston RG, Kuhn SL, editors. Thinking Small: Global Perspectives on Microlithization. Arlington: American Anthropological Association; 2002. p. 117-132.

**Oznachennoye-1**

(Fig.9-32)

*Vasil'ev SA, Kuzmin YV, Orlova LA, Dementiev VN. Radiocarbon-based chronology of the Paleolithic in Siberia and its relevance to the peopling of the New World. Radiocarbon. 2002;44(2):503-530.

Graf KE. Uncharted Territory: Late Pleistocene Hunter-Gatherer Dispersals in the Siberian Mammoth-Steppe: University of Nevada, Reno; 2008.

**Tashtylik-4**

(Fig.9-33)

*Kuzmin YV, Orlova LA. Radiocarbon chronology of the Siberian Paleolithic. Journal of World Prehistory. 1998;12(1):1-53.

Graf KE. Uncharted Territory: Late Pleistocene Hunter-Gatherer Dispersals in the Siberian Mammoth-Steppe: University of Nevada, Reno; 2008.

**Kokorevo-1**

(Fig.9-34)

*Abramova ZA. Paleolithic of the Yenisei: The Kokorevo Culture (in Russian). Novosibirsk: Nauka Publ. 1979.

Graf KE. Hunter–gatherer dispersals in the mammoth-steppe: technological provisioning and land-use in the Enisei River valley, south-central Siberia. Journal of Archaeological Science. 2010;37(1):210-223.

Derevianko AP, Powers WR, Shimkin DB. The Paleolithic of Siberia: New Discoveries and Interpretations (I.P. Larichva, Trans.). Novosibirsk: Institute of Archaeology and Ethnography, Siberian Division, Russian Academy of Sciences; 1998a.

**Novoselovo-7**

(Fig.9-35)

*Lisitsyn NF. The Late Paleolithic of Chulym-Yenisei region (in Russian). St Petersburg: Petersburg Oriental Studies Press. 2000.

*Abramova ZA. Paleolithic of the Yenisei: The Kokorevo Culture (in Russian). Novosibirsk: Nauka Publ. 1979.

Graf KE. Uncharted Territory: Late Pleistocene Hunter-Gatherer Dispersals in the Siberian Mammoth-Steppe. PhD dissertation, University of Nevada, Reno, 2008.

**Afontova Gora**

(Fig.9-36)

*Drozdov NI, Artem’ev EV. New Achievements in the Study of the Paleolithic of Afontova Gora (in Russian). Moscow: INQUA. 1997.

Graf KE. Hunter–gatherer dispersals in the mammoth-steppe: technological provisioning and land-use in the Enisei River valley, south-central Siberia. Journal of Archaeological Science. 2010;37(1):210-223.

Derevianko AP, Powers WR, Shimkin DB. The Paleolithic of Siberia: New Discoveries and Interpretations (I.P. Larichva, Trans.). Novosibirsk: Institute of Archaeology and Ethnography, Siberian Division, Russian Academy of Sciences; 1998a.
